# Smarcal1 promotes double-strand-break repair by nonhomologous end-joining

**DOI:** 10.1093/nar/gkv621

**Published:** 2015-06-18

**Authors:** Islam Shamima Keka, Mohiuddin, Yuko Maede, Md Maminur Rahman, Tetsushi Sakuma, Masamitsu Honma, Takashi Yamamoto, Shunichi Takeda, Hiroyuki Sasanuma

**Affiliations:** 1 Department of Radiation Genetics, Kyoto University, Graduate School of Medicine, Yoshida Konoe, Sakyo-ku, Kyoto 606-8501, Japan; 2 Department of Mathematical and Life Sciences, Graduate School of Science, Hiroshima University, Higashi-Hiroshima 739-8526, Japan; 3 Division of Genetics and Mutagenesis, National Institute of Health Sciences, 1-18-1 Kamiyoga, Setagaya-ku, Tokyo 158-8501, Japan

## Abstract

Smarcal1 is a SWI/SNF-family protein with an ATPase domain involved in DNA-annealing activities and a binding site for the RPA single-strand-DNA-binding protein. Although the role played by Smarcal1 in the maintenance of replication forks has been established, it remains unknown whether Smarcal1 contributes to genomic DNA maintenance outside of the S phase. We disrupted the *SMARCAL1* gene in both the chicken DT40 and the human TK6 B cell lines. The resulting *SMARCAL1^−/−^* clones exhibited sensitivity to chemotherapeutic topoisomerase 2 inhibitors, just as nonhomologous end-joining (NHEJ) null-deficient cells do. *SMARCAL1^−/−^* cells also exhibited an increase in radiosensitivity in the G_1_ phase. Moreover, the loss of Smarcal1 in NHEJ null-deficient cells does not further increase their radiosensitivity. These results demonstrate that Smarcal1 is required for efficient NHEJ-mediated DSB repair. Both inactivation of the ATPase domain and deletion of the RPA-binding site cause the same phenotype as does null-mutation of Smarcal1, suggesting that Smarcal1 enhances NHEJ, presumably by interacting with RPA at unwound single-strand sequences and then facilitating annealing at DSB ends. *SMARCAL1^−/−^*cells showed a poor accumulation of Ku70/DNA-PKcs and XRCC4 at DNA-damage sites. We propose that Smarcal1 maintains the duplex status of DSBs to ensure proper recruitment of NHEJ factors to DSB sites.

## INTRODUCTION

Smarcal1 (a SWI/SNF-related, matrix associated, actin-dependent regulator of chromatin a-like 1) is a SWI/SNF family protein that carries an ATPase domain and the binding site for replication protein A (RPA), the single-strand-DNA-binding protein (1,2). Smarcal1 is an ATP-driven annealing helicase that catalyzes the formation of double-strand DNA from complementary single-strand DNA strands associated with RPA. Thus, Smarcal1 could counteract unwinding by DNA helicases at DSB sites. The annealing activity is stimulated by substrate DNA containing both single-strand and double-strand regions, such as a chicken foot structure (3). Mutations in the *SMARCAL1* gene cause a rare autosomal recessive disease, Schimke immuno-osseous dysplasia (SIOD), which is characterized by short stature, kidney disease and a severely compromised immune system (4–7). Phenotypic analysis of Smarcal1-depleted cells suggests that Smarcal1 stabilizes replication forks when cells are exposed to aphidicolin, hydroxyurea and camptothecin (a topoisomerase 1 poison) (1,2,8–10).

The two major double-strand-break (DSB) repair pathways, homologous recombination (HR) and nonhomologous end-joining (NHEJ) (11–13) significantly contribute to cellular tolerance to anti-malignant therapies. First, both pathways contribute to cellular tolerance to radiotherapy, HR in the S to G_2_ phases and NHEJ throughout the cell cycle. Second, HR plays the dominant role in repairing DSBs generated during DNA replication by chemotherapeutic agents such as camptothecin and poly[ADP ribose]polymerase inhibitor (olaparib). These chemotherapeutic agents cause the accumulation of single-strand breaks, which are converted by DNA replication to DSBs called one-end breaks. These DSBs are repaired by HR but not by NHEJ (14–16). Third, NHEJ plays the dominant role in repairing DSBs caused by chemotherapeutic topoisomerase 2 inhibitors such as ICRF193 and etoposide (15,17). Measuring the sensitivity of gene-disrupted cells to various anti-malignant therapies allows us to define the role of the gene in HR, NHEJ or both. In addition to the above, the capability of canonical NHEJ is evaluated by examining the V(D)J recombination of Immunoglobulin (Ig) V genes, which requires a collaboration between NHEJ and V(D)J recombinase encoded by the recombination-activating-genes 1 and 2 (Rag1/Rag2) (18–20).

Canonical NHEJ is initiated by associating a Ku70/Ku80 heterodimer with DSB sites. Ku70/Ku80 associates preferentially with duplex DNA ends, rather than with DSBs carrying single-strand tails generated by exonucleases or DNA helicases (21–24). Ku70/Ku80 forms a complex with DNA-dependent-protein-kinase catalytic subunit (DNA-PKcs), leading to the activation of DNA-PKcs at DSB sites (25–27). DNA-PKcs phosphorylates a number of substrates, including itself (28–31). Ligase4 (Lig4) completes DSB repair in collaboration with the essential co-factors, XLF and XRCC4, which form clamp-like structures along duplex DNA (32–35). If canonical NHEJ does not perform DSB repair, non-canonical end-joining such as microhomology-mediated alternative end-joining (MMEJ) repairs DSBs, though less efficiently than canonical NHEJ, causing deletion near the DSB sites (36,37).

We disrupted the *SMARCAL1* gene in the chicken DT40 and human B lymphoblastoid TK6 cell lines (38,39). The resulting *SMARCAL1^−/−^* clones exhibited sensitivity to camptothecin, suggesting that Smarcal1 plays a role in DNA replication, as indicated previously (9,10). Remarkably, Smarcal1 is also required for efficient NHEJ in human as well as in chicken cells. This conclusion is in agreement with the fact that SIOD patients exhibit reduced V(D)J recombination products in peripheral lymphocytes as well as increased chromosomal breakage (40,41). We propose that the decreased efficiency of NHEJ in V(D)J recombination as well as the compromised maintenance of replication fork progression result in severe lymphocytopenia in SIOD patients (4,40,41).

## MATERIALS AND METHODS

### Cell clones

All the clones used in this study are summarized in Table 1.

**Table 1.** Panel of cell lines used in this study

Genotype	Parental Cell Line	Markers genes	References
*SMARCAL1^−/−^*	DT40	*hisD, puro^R^*	*
*SMARCAL1^Δ30/−^*	DT40	*hisD*	*
*KU70^−/−^*	DT40	*bsr^R^, puro^R^*	(55)
*BRCA2^−/−^*	DT40	*hygro^R^, hisD*	(46)
*KU70^−/−^/SMARCAL1^Δ30/−^*	DT40	*hisD, bsr^R^, puro^R^*	*
*SMARCAL1^−/−^*	TK6-derived TSCER2 and TSCE5	*puro^R^, hygro^R^*	* T
*LIG4^−/−/−^*	TK6-derived TSCER2 and TSCE5	*puro^R^, neo^R^*	* C
*RAD54^−/−^*	TK6-derived TSCER2	*puro^R^, neo^R^*	* T
*SMARCAL1^−/−^/LIG4^−/−/−^*	TK6-derived TSCE5	*puro^R^, neo^R^, hygro^R^*	* T/C
*DNA-PKcs^−/−^*	TK6-derived TSCE5	*neo^R^, hisD*	* C
*SMARCAL1^−/−^/DNA-PKcs^−/−^*	TK6-derived TSCE5	*neo^R^, hisD*	* T/C

* = This study; T = TALEN; C = CRISPR.

### Cell culture

DT40 and TK6 cells were cultured in the same manner as described previously (39,42).

### Generation of *SMARCAL1^−/−^* DT40 cells

*SMARCAL1* gene disruption constructs were generated from genomic polymerase chain reaction (PCR) products combined with histidinol dehydrogenase (*hisD*) and puromycin-resistance (*puro^R^*) marker genes. Genomic DNA from *wild-type* cells was amplified using the F1 and R1 primers for the 5′-arm and the F2 and R2 primers for the 3′-arm. The amplified 3′-arm PCR product was subcloned into pCR2.1-TOPO vector (Invitrogen, US). The 5′-arm PCR product harboring the *Sac*I and *Bam*HI sites at the 5′- and 3′-ends, respectively, was cloned into the *Sac*I and *Bam*HI sites of the pCR2.1-TOPO vector carrying the 3′-arm. The *Bam*HI fragment containing either the *hisD* or *puro^R^* gene was cloned into the *Bam*HI site between the 3′-arm and the 5′-arm in the pCR2.1-TOPO vector. To generate *SMARCAL1^−/−^* cells, the *SMARCAL1* gene-disruption constructs carrying *hisD* and *puro^R^* were linearized, using the *Not*I restriction enzyme, and sequentially transfected by electroporation (Bio-Rad, US). A 0.5 kb probe was generated by PCR of genomic DNA using primers F3 and R3 for Southern blot analysis. The genomic DNA of the candidate clones was digested with *Sma*I and *Xho*I for Southern blot analysis. The gene disruption was confirmed by RT-PCR, using primers F4 and R4. When generating *SMARCAL1^+/−^* clones from *wild-type* cells the targeting efficiency was 20% (4/20), while that of generating *SMARCAL1^−/−^* cells from *SMARCAL1^+/−^* cells was 1.4% (1/71). All primers used here are shown in Supplemental Table S1.

### Generation of *SMARCAL1^Δ30/−^* and *KU70^−/−^/SMARCAL1^Δ30/−^* DT40 cells

The intact allele of the *SMARCAL1^+/−^* cell was targeted for deleting the first 30 amino acids, which domain is responsible for RPA binding (10). To generate the *Δ30* construct, the 5′-arm was amplified from genomic DNA using the *Not*I-tagged F5 and the *Bam*HI-tagged R5, and the 3′-arm was amplified using the *Bam*HI-tagged F6, which was designed from 90 bases downstream of start codon ATG in exon1, and the *Sal*I-tagged R6. In the Zero Blunt TOPO vector (Invitrogen, US), the left and right arms were cloned at the site of *Not*I-*Bam*HI and *Bam*HI-*Sal*I, respectively. The selection-marker gene, puromycin resistance (*puro^R^*) flanked by *loxP* sequences, was then inserted into the *Bam*HI site between the left and right arms. The resulting *Δ30-puro^R^* construct was transfected into the *SMARCAL1^+/−^* and *KU70^−/−^/SMARCAL1^+/−^* cells. The *puro^R^* gene was popped out by transient expression of cre-recombinase, resulting in the generation of *SMARCAL1^Δ30/−^ and KU70^−/−^/SMARCAL1^Δ30/−^* DT40 cells. To confirm the gene disruption by RT-PCR, the primer set F4 and R4 was used.

### Generation of human *SMARCAL1^−/−^* and *SMARCAL1^−/−^/LIG4^−/−/−^* TK6 B cells

To generate a pair of TALEN expression plasmids against the *SMARCAL1* gene, we used a Golden Gate TALEN kit and a TAL effector kit (Addgene, US) (43,44). The TALEN target sites are shown in Supplementary Figure S2. The gene-targeting constructs were generated from the genomic DNA of TK6 cells by amplifying with primers *Xho*I-flanked F7 and *Nhe*I-flanked R7 for the 5′-arm and *Not*I-flanked F8 and *Hind*III-flanked R8 for the 3′-arm. The 5′-arm PCR products were cloned into the *Xho*I and *Nhe*I sites found upstream of the *puro^R^* and *hygro^R^* marker genes of the DT-ApA/puro and DT-ApA/hygro vectors, respectively. The 3′-arm PCR products were cloned into the *Not*I and *Hind*III sites found downstream of the *puro^R^* and *hygro^R^* marker genes of the DT-ApA/puro and DT-ApA/hygro vectors, respectively. 6 μg TALEN-expression plasmids and 2 μg gene-targeting vectors were transfected into 4×10^6^ TK6 cells using the Neon Transfection System (Life Technologies, US) with 3X pulse at 1350 V and with 10 ms pulse width. After electroporation, cells were released into 20 ml drug-free medium containing 10% horse serum. Forty-eight hours later, cells were seeded into 96-well plates with both hygromycin and puromycin antibiotics for two weeks. The genomic DNAs of the isolated clones resistant to both hygromycin and puromycin were digested with *Xba*I for Southern blot analysis. A 0.6 kb probe was generated by PCR of genomic DNA using primers F9 and R9. The loss of Smarcal1-protein expression was confirmed by western blot analysis (Supplementary Figure S2B). The efficiency of generating *SMARCAL1^−/−^*clones from *wild-type* cells was 100% (3/3). The method for generating *LIG4^−/−/−^* and *RAD54^−/−^* TK6 cells is described in the Supplemental Materials and Methods. *SMARCAL1^−/−^/LIG4^−/−/−^* clones were generated by disrupting the *LIG4* gene in the *SMARCAL1^−/−^* cells. Gene-targeting efficiency was 10% (2/20).

### Generation of human *DNA-PKcs^−/−^* and *SMARCAL1^−/−^/ DNA-PKcs^−/−^* TK6 B cells

To disrupt the *DNA-PKcs* gene, we designed a guide RNA targeting the 32^nd^ exon using the Zhang CRISPR tool (45) and gene-targeting constructs. The CRISPR-target site is depicted in Supplementary Figure S3E. The gene-targeting constructs were generated using SLiCE (Seamless Ligation Cloning Extract). The genomic DNA was amplified with primers F19 and R19 from the *DNA-PKcs*-gene locus and the PCR product was used as template DNA for amplifying the 5′- and 3′-arms. The 5′-arm was amplified using primers F20 and R20 and the 3′-arm was amplified using primers F21 and R21, where each primer shared 20-base pair-end homology with the insertion site of the vector. Both vectors, DT-ApA/neo and DT-ApA/his, were linearized with *Afl*II and *Apa*I. All the fragments of the vectors and inserts were purified using a qiaquick gel extraction kit (QIAGEN, Netherlands). The gene-targeting constructs were generated in a single reaction mixture containing DT-ApA/neo or DT-ApA/his vectors, 5′- and 3′-arms, and 2×SLiCE buffer (Invitrogen, US) and incubated for 30 min at room temperature. 6 μg of CRISPR and 2 μg of each gene-targeting vector were transfected into 4×10^6^ TK6 cells using the Neon Transfection System (Life Technologies, US). After electroporation, cells were released into 20 ml drug-free medium containing 10% horse serum. Forty-eight hours later, cells were seeded into 96-well plates for selection with both neomycin and histidinol antibiotics for two weeks. The gene disruption was confirmed by RT-PCR using primers F22 and R22, and by western blot analysis with anti-DNA-PKcs antibody (Supplementary Figure S3F). The targeting efficiency of generating *DNA-PKcs^−/−^* clones from *DNA-PKcs^+/+^* cells was 90% (9/10). The targeting efficiency of generating *SMARCAL1^−/−^/DNA-PKcs^−/−^* clones from *SMARCAL1^−/−^/DNA-PKcs^+/+^* cells was 100% (2/2).

### Generation of *SMARCAL1^−/−^* cells reconstituted with *SMARCAL1^WT^, SMARCAL1^R764Q^* or *SMARCAL1^Δ30^* transgene

The *SMARCAL1* cDNA was bought from the Kazusa DNA research institute (Chiba, Japan). The *SMARCAL1^R764Q^* cDNA was obtained by site-directed mutagenesis of *SMARCAL1^WT^* (*wild-type SMARCAL1*) cDNA using primers F18 and R18. The *SMARCAL1^Δ30^* cDNA with the first 30 amino acids deleted was generated from *SMARCAL1^WT^* cDNA by PCR using primers F10 and R10. The *SMARCAL1^WT^, SMARCAL1^R764Q^* and *SMARCAL1^Δ30^* transgenes were cloned into pMSCV-IRES-GFP retroviral expression vector (Clontech, US). The newly engineered retroviral expression vector was co-transfected into human 293T cells with a helper plasmid (pClampho, US) to produce a viral supernatant, which was collected after 24 hours and used to infect the *SMARCAL1^−/−^* cells. The efficiency of infection was assessed by quantifying the number of cells expressing GFP using flow-cytometric analysis (LSRFortessa, BD Biosciences, US). The cells expressing GFP were enriched using a cell sorter (FACSAria III, BD Biosciences, US) and seeded into 96 well plates to isolate single colonies. The expression level of the transgenes in the *SMARCAL1^−/−^* cells was measured by western blot (Figure 5D).

**Figure 1.**
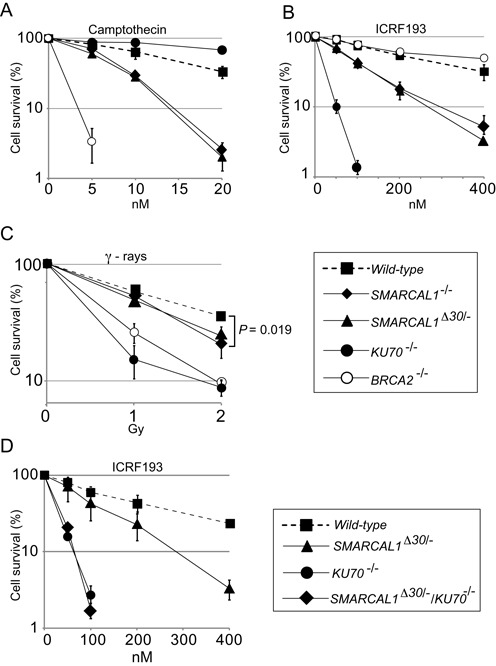
Smarcal1-deficient DT40 cells are sensitive to ICRF193 and camptothecin. Clonogenic-cell-survival assay following exposure of the indicated genotypes to DNA-damaging agents (**A**–**D**). The *x*-axis represents the dose of the indicated DNA-damaging agent on a linear scale; the *y*-axis represents the survival fraction on a logarithmic scale. Error bars show the SD of the mean for three independent assays. The *P*-value of (C) was calculated by Student's *t*-test or two-sample *t*-test for IR sensitivity at 2 Gy.

**Figure 2.**
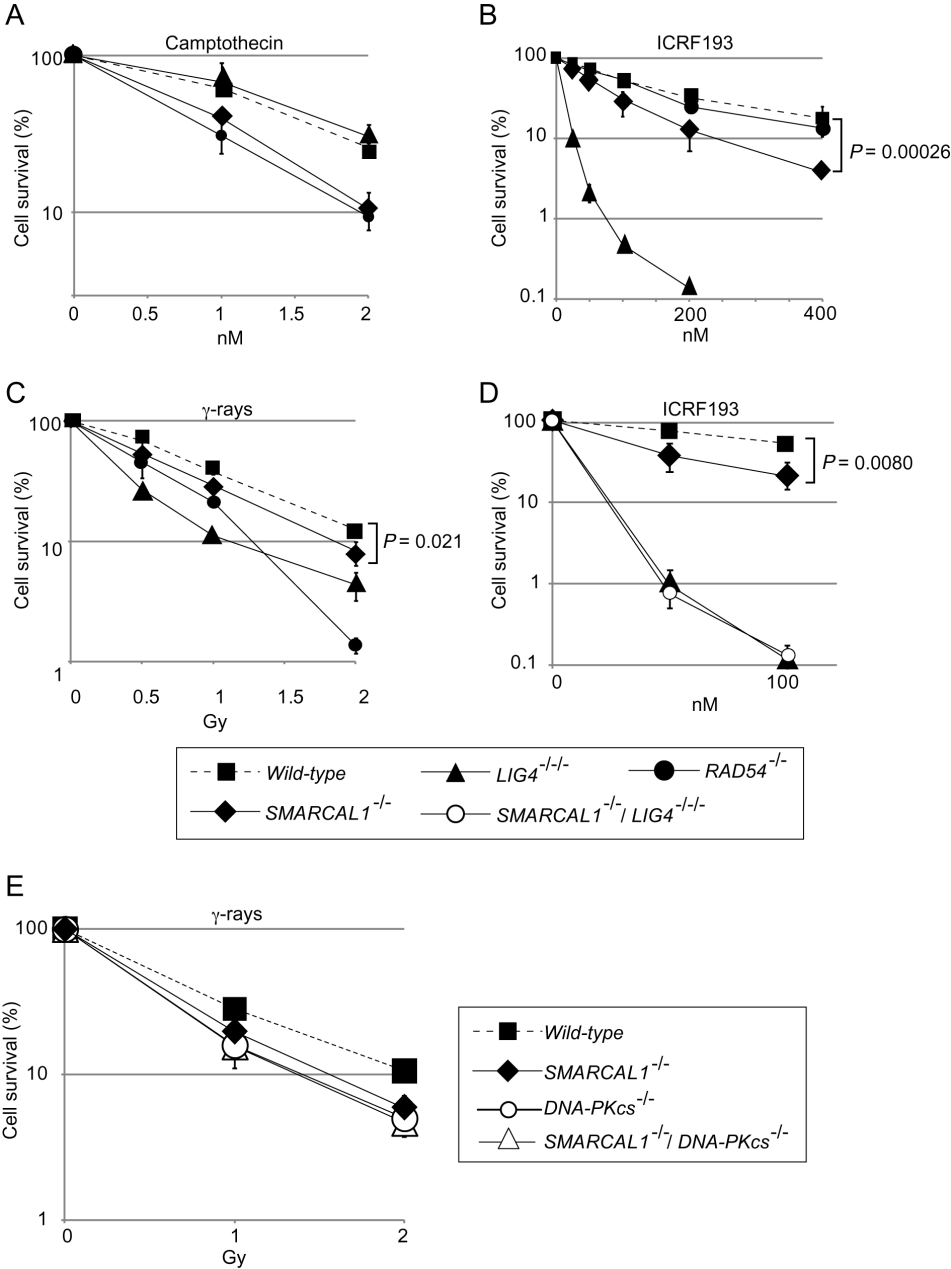
Sensitivity of human *SMARCAL1^−/−^, SMARCAL1^−/−^/LIG4^−/−/−^* and *SMARCAL1^−/−^/DNA-PKcs^−/−^* TK6 B cells to ICRF193 and γ-rays. (**A**–**E**) Cellular sensitivity is shown as in Figure 1. Error bars show the SD of the mean for three independent assays. *P*-values were calculated by Student's *t*-test.

**Figure 3.**
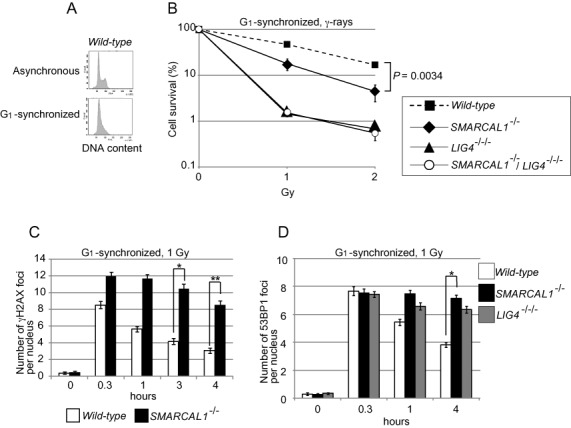
Repair of γ-ray induced DSBs at the G_1_ phase in TK6 cells. (**A**) DNA content of G_1_-synchronized TK6 cells. (**B**) Cellular sensitivity of G_1_-synchronized cells to **γ**-rays, shown as in Figure 1. Error bars indicate the SD of the mean for three independent assays. *P*-value was calculated by Student's *t*-test. (**C**) Histogram representing the γH2AX subnuclear foci of G_1_ cells after irradiation with 1 Gy **γ**-rays. The *x*-axis represents time after γ-irradiation (time zero); the *y*-axis represents the average number of γH2AX foci in individual cells. The nuclei of 100 morphologically intact cells were analyzed at each time point in individual experiments. The experiment was performed at least three times, with the averages presented with SD and *P*-values. Asterisks indicate statistical significance; *P = 0.0055 and **P = 0.00020. (**D**) Histogram representing the 53BP1 subnuclear foci of G_1_ cells, as shown in (C). The experiment was performed at least three times, with averages presented with SD and *P*-value. Asterisks indicate statistical significance; *p = 6.3×10^−5^.

**Figure 4.**
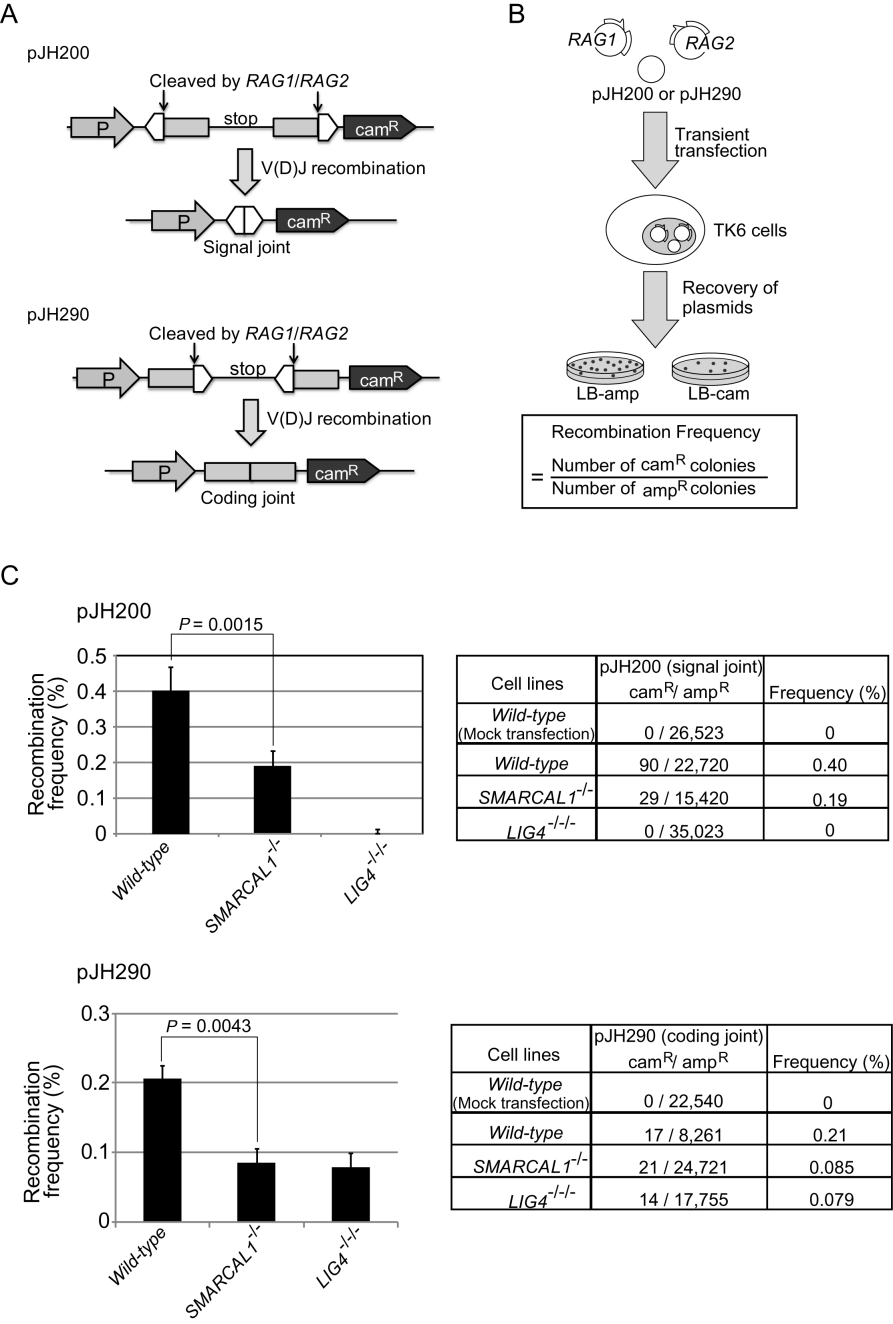
The loss of Smarcal1 reduces the efficiency of V(D)J recombination without compromising its fidelity. (**A**) The structure of two episomal V(D)J-recombination substrates, pJH200 and pJH290, and their recombination products. Open triangles and closed boxes represent recombination signals (RSs) and V(D)J-coding sequences, respectively. Cam^R^ = chloramphenicol-resistance gene; P = promoter. (**B**) Schematic representation of the experimental method for the V(D)J-recombination assay. Frequency of recombination was calculated by dividing the number of rearranged products (the number of cam^R^ colonies) by the number of recovered plasmids (the number of ampicillin-resistant [amp^R^] colonies). (**C**) Recombination frequency of TK6 cells carrying the indicated genotypes. Data shown are the means of more than three experiments. Error bars indicate SD of more than three independent experiments. *P*-value was calculated by Student's *t*-test. The total number of ampicillin- and chloramphenicol-resistant colonies is shown in the right panel. Supplemental Table S2 shows the nucleotide sequences of coding joints associated with deletion events.

**Figure 5.**
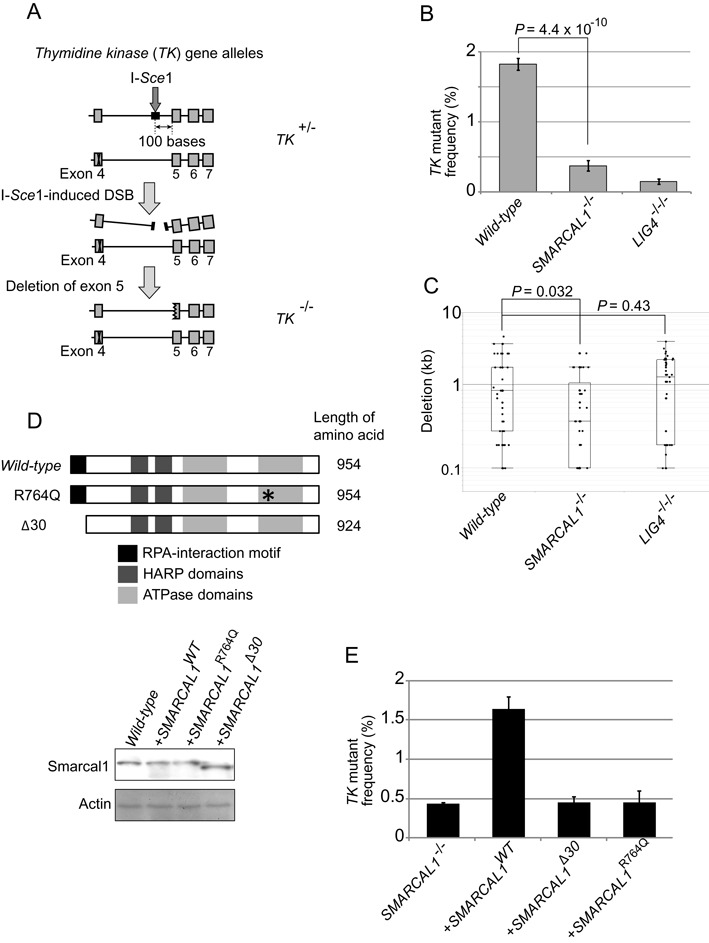
The fidelity of end-joining in *SMARCAL1* mutant cells. (**A**) Schematic diagram showing DSB-repair events that repair I-*Sce*1-induced DSBs in the endogenous thymidine kinase (*TK*) locus. *TK^+/−^* cells carry an I-*Sce*1 site in intronic sequences of the *wild-type TK* allele and a mutation in exon4 of the mutant *TK* allele. DSB repair associated with deletion in exon5 coding sequences would yield *TK^−/−^* clones from *TK^+/−^* cells. The number of *TK^−/−^* clones was measured by counting the number of trifluorothymidine (TFT)-resistant colonies. (**B**) Histogram representing the frequency of DSB-repair events (*y*-axis) in the indicated genotypes (*x*-axis). Error bars indicate SD of more than three independent experiments. *P*-value was calculated by Student's *t*-test. (**C**) Box plot representing the length of nucleotide deletion (*y*-axis) in the indicated genotypes (*x*-axis). PCR was performed from genomic DNA isolated from at least 50 TFT-resistant clones of each genotype, as shown in Supplementary Figure S6. (**D**) Schematic representation of the structure of *wild-type*, R764Q and Δ30 Smarcal1 proteins. *SMARCAL1^−/−^* TK6 cells were reconstituted with *SMARCAL1^WT^, SMARCAL1^Δ30^* or *SMARCAL1^R764Q^* transgene. Western blot analysis for the expression of individual transgenes in *SMARCAL1^−/−^* cells. β-actin was used as a loading control. (**E**) Histogram representing the frequency of TFT-resistant colonies (*y*-axis) in the indicated genotypes (*x*-axis). Error bars indicate SD of more than three independent experiments.

### Colony-survival assay

To measure sensitivity, cells were treated with camptothecin (Topogen, Inc, US) and ICRF193 (Funakoshi, Japan) (17) and irradiated with ionizing radiation (^137^Cs). Cell sensitivity to these DNA-damaging agents was evaluated by counting colony formation in methylcellulose plates as described previously (46,47).

### Cell-cycle synchronization

Cells were synchronized by centrifugal-counter-flow elutriation (Hitachi Koki, Japan). The cell suspension (∼5×10^7^ TK6 cells) was loaded at a flow rate of 15 ml/min into an elutriation chamber rotating at 2000 rpm. Cell synchrony was confirmed by FACS analysis (LSRFortessa, BD Biosciences, US).

### Immunostaining and microscopic analysis

Cells were fixed with 4% paraformaldehyde (Nacalai Tesque, Japan) for 10 min at room temperature and permeabilized with 0.5% TritonX-100 (Sigma, St. Louis, US) for 20 min. To exclude S-phase cells from the count shown in Figure 3C and D, a Click-iT EdU imaging kit (Alexa594, Invitrogen, US) was used. Images were taken with a confocal microscope (TCS SP8, Leica Microsystems, Germany) and a BX-61 microscope (Olympus, Japan).

### V(D)J-recombination assay

The V(D)J-recombination assay was performed as described previously (48). Briefly, 4×10^6^ TK6 cells were transfected (Neon, Life Technologies, US) with 600 ng of circular pJH200 or pJH290, 5.4 μg of *RAG1*- and 6.6 μg of *RAG2*-expression vector. The extrachromosomal plasmids were recovered from cells after 48 hours using a modified Hirt extraction method (49). The mixture of 15 μl of ElectroMAX^TM^ DH10B^TM^ (Life Technologies, US) competent bacteria and 300 ng (range of 100–500 ng) of recovered plasmids were added to an electroporation cuvette (0.1 cm gap) and incubated on ice for 10 min. The bacteria were then electroporated at 1.8 kV, 200 Ω and 25 uF for 2 seconds using a Gene PulserII (Bio-Rad, US). Pre-warmed SOC media was added to the bacteria and the reaction was incubated at 37°C for 2 hours. The reaction was plated on LB-agar plates containing 100 μg/ml ampicillin and 10 μg/ml chloramphenicol and incubated for 16–24 hours at 37°C. The ampicillin+chloramphenicol-resistant plasmids were isolated and subjected to *Apa*LI digestion to examine the fidelity of the signal joints. Digestion of original pJH200 plasmid yields a 4.3 kb band, while that of the correct recombination products yields 3.5 kb + 0.8 kb bands due to the newly generated *Apa*LI site. Signal-joint and coding-joint sequences were analyzed using the sequencing primer R17 (50).

### NHEJ assay of I-*Sce*1-induced DSBs

A TK6-derived line that is heterozygous for point mutation in exon4 of the thymidine-kinase gene (*TK^+/−^*) was used to measure the frequency of NHEJ events as described previously (51,52). To measure the length of deletion in DSB-repair products formed in *wild-type* and *SMARCAL1^−/−^* cells, primers Fa and Ra were used. To measure the length of the deletion formed in the *LIG4^−/−/−^* cells, primers Fa and Rb were used.

### Chromatin fractionation and chromatin immunoprecipitation

A Subcellular Protein Fractionation Kit from Thermo Scientific (78840) was used for chromatin fractionation. Expression plasmids for a TALEN and I-*Sce*1 were transfected into TK6 cells using the Neon Transfection System. After 20 hours, transfected cells were analyzed by western blotting and ChIP. ChIP was performed as described previously (53), with some modifications. Briefly, samples were sonicated to generate DNA fragments of <500 bp. The antibody was incubated with Dynabeads Protein G for 3 hours at 4°C. Sheared chromatin was centrifuged at 15000 rpm for 15 min at 4°C. After centrifugation, supernatants were incubated with antibody-protein G conjugates for 3 hours at 4°C. The conjugated beads were washed thoroughly with IP buffer-140, IP buffer-500, IP buffer-750, LiCl/detergent and TE. Real-time PCR was carried out as described previously (53). Sequences of primers are given in Supplemental Table S1.

### Antibodies

Anti-γH2AX mouse monoclonal (1:1000, Millipore, US); anti-TP53BP1 rabbit polyclonal (1:100, Sigma, US); alexa fluor 488-conjugated anti-mouse IgG (1:1000, Molecular Probes); alexa fluor 488–conjugated anti-rabbit IgG (1:1000, Molecular Probes); anti-Smarcal1 rabbit polyclonal (ab154226, abcam, UK); anti-XRCC4 goat polyclonal (C-20, Santa Cruz, US); anti-Ku70 mouse polyclonal (#GTX70270, Gene Tex, US); anti-DNA-PKcs mouse monoclonal (ab1832, abcam, UK).

## RESULTS

### *SMARCAL1*-deficient cells are sensitive to DNA-damaging agents

To analyze the role of Smarcal1 in the DSB-repair pathway, we disrupted the *SMARCAL1* gene and generated *SMARCAL1^−/−^* DT40 cells (Supplementary Figures S1A and S1C). Moreover, to selectively analyze the function of the Smarcal1-RPA interaction, we generated *SMARCAL1^Δ30/−^* DT40 mutant cells by deleting the N-terminal region encoding the RPA-binding site of the endogenous *SMARCAL1* gene (9) (Supplementary Figures S1D and S1E). To define the role played by Smarcal1 in various DNA-repair processes, we measured cellular responses to exogenous DNA damages. *SMARCAL1^−/−^* and *SMARCAL1^Δ30/−^* DT40 cells exhibited increased sensitivities to various DNA-damaging agents, including camptothecin and ICRF193 (Figures 1A and B). The sensitivity profile of the *SMARCAL1^Δ30/−^* cells was very similar to that of the *SMARCAL1^−/−^* cells, suggesting that the physical association of Smarcal1 with RPA is essential for its DNA-damage response (9). The elevated sensitivity to camptothecin supports the idea that Smarcal1 helps to prevent replication forks from replication collapse at one-end breaks, as indicated previously (1,2,8–10,54). NHEJ-deficient *KU70^−/−^* cells (55), but not HR-deficient *BRCA2^−/−^*cells (46), showed a hypersensitivity to ICRF193 (Figure 1B). We further analyzed the functional relationship between Smarcal1 and Ku70 by generating *KU70^−/−^/SMARCAL1^Δ30/−^* double-mutant cells. The double-mutant cells showed virtually the same ICRF193 sensitivity as did the *KU70^−/−^* single mutant (Figure 1D), indicating that Smarcal1 is epistatic to Ku70.

To investigate the role of Smarcal1 in human cells, we disrupted the *SMARCAL1* gene in the human TK6 B cell line using a TALEN pair combined with gene-disruption constructs (Supplementary Figures S2A and S2B). We also generated both *LIG4^−/−/−^* (Supplementary Figures S3A and S3B) and *RAD54^−/−^* TK6 clones (Supplementary Figures S3C and S3D) as controls deficient in NHEJ and HR, respectively. In addition, we generated *DNA-PKcs^−/−^* TK6 clones (Supplementary Figures S3E and S3F). The TK6 cell line has been widely used by the governments of developed countries to detect environmental mutagens due to the very stable phenotype and karyotype of its cells (56,57). The *SMARCAL1^−/−^*cells proliferated with kinetics (15 h per cell cycle) and plating efficiency (58%) similar to that of *wild-type* TK6 cells. Like the DT40 mutants, human *SMARCAL1^−/−^* cells were moderately but also significantly sensitive to camptothecin (Figure 2A). Three *SMARCAL1^−/−^* clones and NHEJ-deficient *LIG4^−/−/−^* and *DNA-PKcs^−/−^*, but not HR-deficient *RAD54^−/−^*, were sensitive to ICRF193 (Figure 2B, Supplementary Figures S2C and S4B). Additionally, *LIG4^−/−/−^* and *SMARCAL1^−/−^/LIG4^−/−/−^* cells showed the same sensitivity to ICRF193 (Figure 2D). Likewise, *DNA-PKcs^−/−^* and *SMARCAL1^−/−^/DNA-PKcs^−/−^* cells showed the same sensitivity to ICRF193 (Supplementary Figure S4B). We thus conclude that Smarcal1 promotes the canonical NHEJ pathway in both DT40 and TK6 cell lines.

### Human *SMARCAL1^−/−^* cells are defective for DSB repair in the G_1_ phase

*SMARCAL1^−/−^* DT40 and TK6 cells were significantly radiosensitive (Figures 1C and 2C and Supplementary Figure S2C). Remarkably, *SMARCAL1^−/−^, DNA-PKcs^−/−^* and *SMARCAL1^−/−^/DNA-PKcs^−/−^* TK6 cells showed very similar radiosensitivity (Figure 2E and Supplementary Figure S4A). To further investigate the role of Smarcal1 in NHEJ, we measured sensitivity to ionizing radiation (IR) in the G_1_ phase (Figure 3A and Supplementary Figure S5A). *SMARCAL1^−/−^* as well as *LIG4^−/−/−^* cells showed a hypersensitivity to IR (Figure 3B). As expected, the *SMARCAL1^−/−^/LIG4^−/−/−^* cells showed the same hypersensitivity to IR as did the *LIG4^−/−/−^* cells in the G_1_ phase (Figure 3B). We then monitored DSB repair kinetics in the G_1_ phase by counting the number of γH2AX and 53BP1 foci over time after exposure to IR. The resolution of 53BP1 and γH2AX foci was significantly delayed in *SMARCAL1^−/−^* cells compared to *wild-type* cells (Figures 3C, D and Supplementary Figure S5B). These results indicate that *SMARCAL1^−/−^* cells are deficient in DSB repair in the G_1_ phase. We therefore conclude that Smarcal1 promotes DSB repair by NHEJ in both human and chicken DT40 cells.

### Deletion of Smarcal1 does not compromise the fidelity of NHEJ

To address the accuracy of individual DSB-repair events, we performed two experiments using TK6 cells, (i) the analysis of V(D)J recombination (Figure 4) and (ii) the repair of I-*Sce*1 induced DSBs by NHEJ (Figure 5).

V(D)J recombination is initiated by the Rag1 and Rag2 recombinase proteins, which introduce DSBs at the recombination signal (RS) (58,59) and complete recombination by collaborating with canonical NHEJ. We used two episomal V(D)J-recombination substrates, pJH200 and pJH290, where the recombinase generates the RS and coding-joint products, respectively (50) (Figure 4A). We transiently transfected expression plasmids encoding *RAG1* and *RAG2* along with either pJH200 or pJH290 into *wild-type* and *SMARCAL1^−/−^* and *LIG4^−/−/−^* cells (Figure 4B). We then recovered the transfected substrate plasmids from the TK6 cells, introduced the plasmids into bacterial cells, and plated them on LB agar plates containing either ampicillin or chloramphenicol. Ampicillin-resistant colonies contained recovered pJH200 or pJH290 plasmids, while colonies resistant to chloramphenicol contained only rearranged pJH200 or pJH290 plasmids. Thus, the frequency of rearrangement can be calculated as the gain of chloramphenicol-resistant (cam^R^) colonies relative to the total number of ampicillin-resistant (amp^R^) colonies. The signal-joint ends are precisely ligated, whereas the coding ends are joined in a process that can involve nucleotide loss or gain in addition to simple ligation (60). We failed to recover any rearranged signal-joint products from *LIG4^−/−/−^* cells, which agrees with the essential role for DNA Ligase4 in signal-joint formation (61,62). The efficiency of recombination in the signal-joint and coding-joint plasmids was decreased 2.3 and 2.4 times, respectively, in *SMARCAL1^−/−^* cells, when compared with *wild-type* cells (Figure 4C). The nucleotide sequence analysis of signal-joint products indicated that only a single product among the 29-analyzed sequences contained one nucleotide deletion in *SMARCAL1^−/−^* cells (data not shown). Analysis of coding joints, on the other hand, showed more frequent deletion events in *SMARCAL1*^−/−^ as well as in *wild-type* cells, with the extent and frequency of deletion being comparable between the two genotypes (Supplemental Table S2). Thus, the loss of Smarcal1 reduces the efficiency of canonical NHEJ without compromising its fidelity.

We next examined individual DSB-repair events in the human TK6 cell line, which is heterozygous (+/-) for the thymidine kinase (*TK*) gene and contains an I-*Sce*1 endonuclease recognition site in the fourth intron of the intact *TK* allele (51) (Figure 5A). If the repair of I-*Sce*1-induced DSBs causes the deletion of more than 100 nucleotides downstream from the I-*Sce*1 site, the deletion would inactivate the fifth exon, leading to the formation of *TK^−/−^* cells, which cells are able to form colonies in the negative-selection media containing trifluorothymidine (TFT) (Figure 5A). We found that the number of TFT-resistant clones was reduced by 8 and 5 times in the *LIG4^−/−/−^* and *SMARCAL1^−/−^* cells, respectively, when compared with *wild-type* cells (Figure 5B). To analyze deletion range and pattern, we isolated at least 50 individual TFT-resistant clones from each genotype. Genomic PCR amplification over the I-*Sce*1 site indicated that deletion was two times shorter in the *SMARCAL1^−/−^* cells and approximately 0.5 times longer in *LIG4^−/−/−^* cells, compared with *wild-type* cells (Figure 5C and Supplementary Figure S6). In summary, we conclude that Smrcal1 promotes NHEJ without affecting its accuracy.

### The RPA-binding domain and the ATPase domains are both required for the promotion of NHEJ by Smarcal1

The phenotypic analysis of *SMARCAL1^Δ30/−^* DT40 cells indicated that the RPA-binding site is essential for Smarcal1 to function in NHEJ. To confirm the relevance of this finding to human cells, we reconstituted the *SMARCAL1^−/−^* TK6 cells with the RPA-binding-site-deficient (*SMARCAL1^Δ30^*) transgene as well as the *wild-type* (*SMARCAL1^WT^*) transgene. The expression level of the Smarcal1 protein in the reconstituted cells was similar to that in the *wild-type* cells (Figure 5D, lower panel). As expected, reconstitution of *SMARCAL1^−/−^* cells with the *SMARCAL1^WT^* transgene normalized the NHEJ-mediated repair of I-*Sce*1-induced DSBs (Figure 5E). The *SMARCAL1^Δ30^* transgene failed to normalize NHEJ, indicating that the RPA-binding site plays a role in promoting NHEJ by Smarcal1 in human cells. Moreover, *SMARCAL1^−/−^* cells and *SMARCAL1^Δ30^* transgene exhibited similar sensitivity to DNA-damaging agent (Supplementary Figure S4C), demonstrating that the RPA-binding site is critical for Smarcal1 DNA repair function. Next, to investigate the role of annealing helicase activity, we introduced a point mutation (Arginine 764 to Glutamine, R764Q) into the ATPase domain of the *SMARCAL1^WT^* transgene, which mutation has no detectable annealing helicase activity (2) and causes a severe form of Schimke immuno-osseous dysplasia (SIOD) (4). Reconstitution with the resulting *SMARCAL1^R764Q^* transgene did not restore the repair of I-*Sce*1-induced DSBs (Figure 5D and E). We therefore conclude that the annealing helicase activity as well as the physical association of Smarcal1 with RPA is required for the promotion of NHEJ by Smarcal1. One possible scenario is that Smarcal1 promotes NHEJ by interacting with unwound DSB ends associated with RPA and facilitate their annealing.

Annealing of double-strand DNA by purified Smarcal1 (2) suggests that Smarcal1 facilitates NHEJ by stabilizing the physical interaction between Ku70/Ku80/DNA-PKcs proteins and DSB ends, thereby activating DNA-PKcs. To test this hypothesis, we analyzed the phosphorylation status of threonine 2609 (63,64) after treatment with the topoisomerase 2 inhibitors ICRF193 (Supplementary Figures S7A and S7B) and etoposide (Supplementary Figure S7C). Strikingly, the phosphorylated threonine 2609 was detectable only in the *wild-type* and not in the *SMARCAL1^−/−^* cells (Supplementary Figures S7B and S7C). The compromised phosphorylation of DNA-PKcs may account for the decreased efficiency of NHEJ, as the substitution of the threonine 2609 site to alanine causes radio-sensitivity and reduced V(D)J recombination efficiency, which sensitivity is less prominent than that of *DNA-PKcs* null-mutant cells (64). We propose that Smarcal1 promotes NHEJ, presumably at an initial step, by facilitating the physical association of Ku70/Ku80/DNA-PKcs proteins and DSB ends.

### Smarcal1 is required for the recruitment of XRCC4 to DNA-damage sites

**Figure 6.**
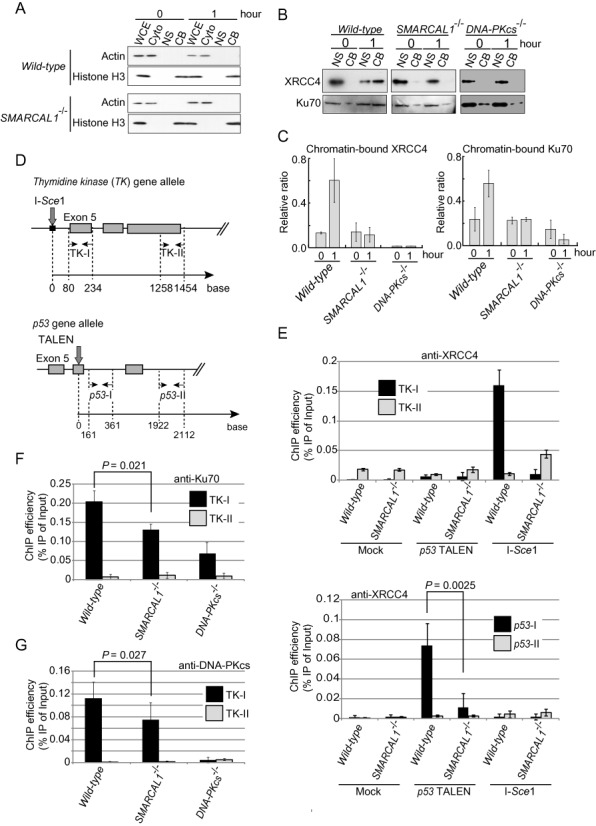
The loss of Smarcal1 results in compromised accumulation of Ku70, DNA-PKcs and XRCC4 at DSB sites. (**A**) Western blot data showing the validation of fractionation of the cytoplasmic (Cyto), nuclear soluble (NS) and chromatin-bound (CB) fractions isolated from the whole-cell extract (WCE). (**B**) Western blot data show the accumulation of XRCC4 (upper panel) and Ku70 (lower panel) in the chromatin-bound fraction after one-hour exposure of cells to ICRF193. (**C**) Histogram showing the quantification of XRCC4 and Ku70 in (B). The *y*-axis represents the amount of the chromatin-bound fraction relative to the total amount of the chromatin-bound fraction plus the nuclear soluble fraction. (**D**) Downward arrows represent the I-*Sce*1- (upper) and the TALEN- (lower) cutting sites in the *TK* and *p53* genes, respectively. Pairs of horizontal opposing arrows indicate the sets of primers for quantitative real-time PCR. (**E**) Histograms represent the accumulation of XRCC4 near the I-*Sce*1-induced DSB in the *TK* locus (upper panel) and near the TALEN-induced DSB at the *p53* locus (lower panel). (**F**) Histogram represents the accumulation of Ku70 near the I-*Sce*1-induced DSB in the *TK* locus. (**G**) Histogram represents the accumulation of DNA-PKcs near the I-*Sce*1-induced DSB in the *TK* locus.

To investigate early and late steps of NHEJ, we monitored the dynamics of Ku70 and XRCC4 proteins, respectively, in the chromatin-bound fraction, following exposure of cells to ICRF193 for one hour (Figure 6). This exposure did not affect the purification of the chromatin-bound or the nuclear-soluble fractions (Figure 6A). Exposure to ICRF193 caused a marked increase in the amounts of both Ku70 and XRCC4 in the chromatin-bound fraction of *wild-type* cells. In marked contrast, accumulations of Ku70 and XRCC4 in the *SMARCAL1^−/−^* as well as *DNA-PKcs^−/−^* cells were, significantly smaller compared with *wild-type* cells (Figure 6B and C).

To validate the significant reduction in the accumulation of XRCC4 near DSB sites, we conducted chromatin immunoprecipitation (ChIP) following transient transfection of empty vector and expression vectors encoding the I-*Sce*1 restriction enzyme and a TALEN towards the *p53* locus (Figure 6D). XRCC4 is supposed to accumulate at the *TK-I* or *p53-I* sites, which are adjacent to the DSB sites, but not at the *TK-II* or *p53-II* sites, which are distant from the DSB sites (Figure 6D). Transient expression of I-*Sce*1 or TALEN caused the accumulation of XRCC4 near the DSB sites in *wild-type* cells, whereas the extent of the accumulation was significantly reduced in *SMARCAL1^−/−^* cells (Figure 6E and Supplementary Figure S7D). We checked the overall expression levels of XRCC4 (Supplementary Figure S7B) to confirm that the deletion of Smarcal1 did not alter the level of protein expression. Thus, Smarcal1 is required for recruitment of XRCC4 to DSB sites.

Like reduced accumulation of Ku70 in the chromatin fraction (Figures 6B and C), the amounts of Ku70 and DNA-PKcs near the I-*Sce*1 site were approximately 30% smaller in *SMARCAL1^−/−^* cells in comparison with *wild-type* cells (Figures 6F and G). In summary, Smarcal1 may promote annealing of DSB ends, which stabilizes complex formation of Ku/DNA-PKcs at DSB sites and fully activates DNA-PKcs. The activation may enhance the recruitment of XRCC4 for the completion of DSB repair by canonical NHEJ.

## DISCUSSION

We herein show that Smarcal1 significantly contributes to canonical NHEJ. Although the role of Smarcal1 during DNA replication has been well established (1,3,8–10,65), it has remained unclear whether Smarcal1 plays a role outside the S phase. We here reveal that Smarcal1 contributes to DSB repair by NHEJ during the G_1_ phase. The role played by Smarcal1 in NHEJ is demonstrated by six points, as follows. First, the loss of Smarcal1 increases cellular sensitivity to ICRF193, which induces DSBs repair only by NHEJ and not by HR (17) (Figures 1B and 2B). Second, null-mutation of *KU70* is epistatic to *SMARCAL1^Δ30^* mutation in DT40 cells (Figure 1D) and null-mutation of *LIG4* is epistatic to *SMARCAL1* null-mutation in TK6 cells (Figure 2D), in terms of cellular tolerance to ICRF193. Third, the loss of Smarcal1 significantly reduces the efficiency of DSB repair in the G_1_ phase in TK6 cells (Figure 3). Fourth, *SMARCAL1* null-mutation in TK6 cells significantly compromises V(D)J recombination (Figure 4) as well as I-*Sce*1-induced DSB repair by NHEJ (Figure 5). Fifth, *SMARCAL1* null-mutation impairs the phosphorylation of DNA-PKcs at threonine 2609 (Supplementary Figures S7B and S7C). Lastly, *SMARCAL1* null-mutation diminishes the accumulation of Ku70, DNA-PKcs and XRCC4 at DNA-damage-induced DSB sites (Figure 6). We therefore conclude that Smarcal1 plays a role in NHEJ.

The molecular mechanisms underlying the severe lymphocytopenia of SIOD patients remain unclear (4,40,41). The lymphocytopenia might result primarily from a severe defect in V(D)J recombination due to the reduced efficiency of NHEJ, according to the following studies. The analysis of V(D)J recombination in peripheral T lymphocytes indicates that the size of the T-cell-antigenic receptor (TCR) repertoire is extremely small in SIOD patients (40). Moreover, the peripheral T lymphocytes of SIOD patients have severely low copy number of episomal circular DNA generated as a consequence of V(D)J recombination and contain signal joints of the T cell receptor genes (40,41). These observations support the following scenario. A moderate defect in NHEJ can cause a very strong decrease in the efficiency of T-cell development in patients, since it requires productive D-J- and V-D-recombination events in both TCRα and TCRβ chain genes in individual thymocytes. The reduced T-cell production by the thymus may be compensated by enhanced proliferation of newly generated peripheral T lymphocytes (66) in SIOD patients, leading to the quick dilution of episomal circular DNA in individual peripheral T lymphocytes. This enhanced proliferation could cause a strong replication stress in SIOD patients, due to their attenuated stabilization of replication forks. In summary, we propose that the severe lymphocytopenia of SIOD patients (4,40,41) is attributable to the significantly reduced efficiency of V(D)J recombination, together with attenuated stabilization of replication forks.

A prominent question is, how does Smarcal1 facilitate the promotion of NHEJ? We have shown that both the loss of the RPA-binding site and the inactivation of ATPase activity in Smarcal1 completely abolished the promotion of NHEJ by Smarcal1 (Figure 5E). Thus, Smarcal1 plays a role in NHEJ by physically interacting with RPA on unwound DSB ends and then facilitating their annealing. The existence of unwound single-stranded sequences at the DSB sites is supported by the presence of RPA foci in the γ-ray irradiated G_1_ phase cells (67). The annealing by Smarcal1 may stabilize the interaction of DSB sites with DNA-PKcs/Ku70/Ku80, since Ku70/Ku80 associates with duplex DSB ends more stably than with DSBs carrying single-strand tails (21–24). The stabilization of DNA-PKcs/Ku70/Ku80 at DSB sites by Smarcal1 is verified by data shown in Figure 6. The following data suggest the important role of Smarcal1 in the functioning of DNA-PKcs. DNA-PKcs/Ku70/Ku80 also interacts with Smarcal1 *in vivo* (68,69). We here show that *DNA-PKcs^−/−^* and *SMARCAL1^−/−^* have an epistatic relationship in cellular tolerance to IR (Figure 2E). We also show that the loss of Smarcal1 inhibits the phosphorylation of DNA-PKcs at threonine 2609 following exposure of cells to the topoisomerase 2 inhibitors (Supplementary Figure S7). These observations suggest that Smarcal1 may be required for DNA-PKcs/Ku70/Ku80 to function appropriately. We also show that the loss of Smarcal1 reduces the recruitment of XRCC4 to DSB sites by several times (Figure 6). Previous studies indicate that DNA-PKcs is necessary for the stabilization of recruited XRCC4 (70,71), which is consistent with our data (Figure 6B). Thus, the effect of Smarcal1 on the recruitment of XRCC4 might be mediated by DNA-PKcs/Ku70/Ku80. We therefore propose that Smarcal1 maintains duplex DNA status at DSB ends by interacting with unwound single-strand DNA associated with RPA and facilitating their annealing. This annealing then stabilizes DNA-PKcs/Ku70/Ku80 at duplex DNA termini, which is essential for the proper accumulation and stabilization of XRCC4 at DNA damage sites. Future studies should clarify the molecular mechanism.

## SUPPLEMENTARY DATA

Supplementary Data are available at NAR Online.

SUPPLEMENTARY DATA
