# Supplementary material for: Smarcal1 promotes double-strand-break repair by nonhomologous end-joining
Source: Nucleic Acids Res. 2015 Jun 18;43(13):6359–72. doi: 10.1093/nar/gkv621 (PMC4513880; doi:10.1093/nar/gkv621)
Supplement: SUPPLEMENTARY DATA [file supp_43_13_6359__index.html]

Smarcal1 promotes double-strand-break repair by nonhomologous end-joining — SUPPLEMENTARY DATA 

# Smarcal1 promotes double-strand-break repair by nonhomologous end-joining

## SUPPLEMENTARY DATA

- SUPPLEMENTARY DATA
