# Supplementary material for: Smarcal1 promotes double-strand-break repair by nonhomologous end-joining
Source: Nucleic Acids Res. 2015 Jun 18;43(13):6359–72. doi: 10.1093/nar/gkv621 (PMC4513880; doi:10.1093/nar/gkv621)
Supplement: SUPPLEMENTARY DATA [file supp_gkv621_SUPPLEMENTALMM_text2.pdf]

Keka, *et al*., Supplemental Figure 1

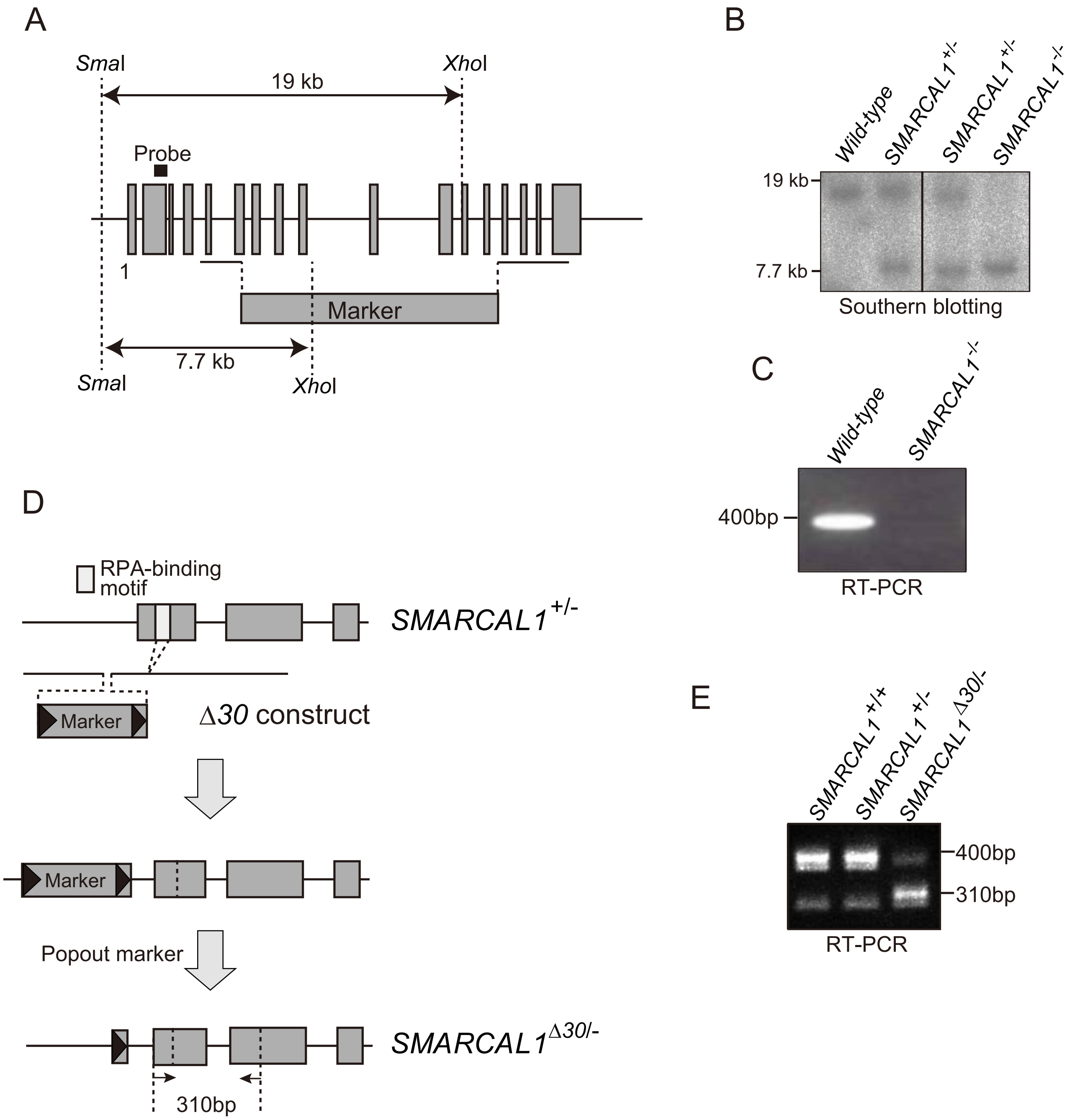

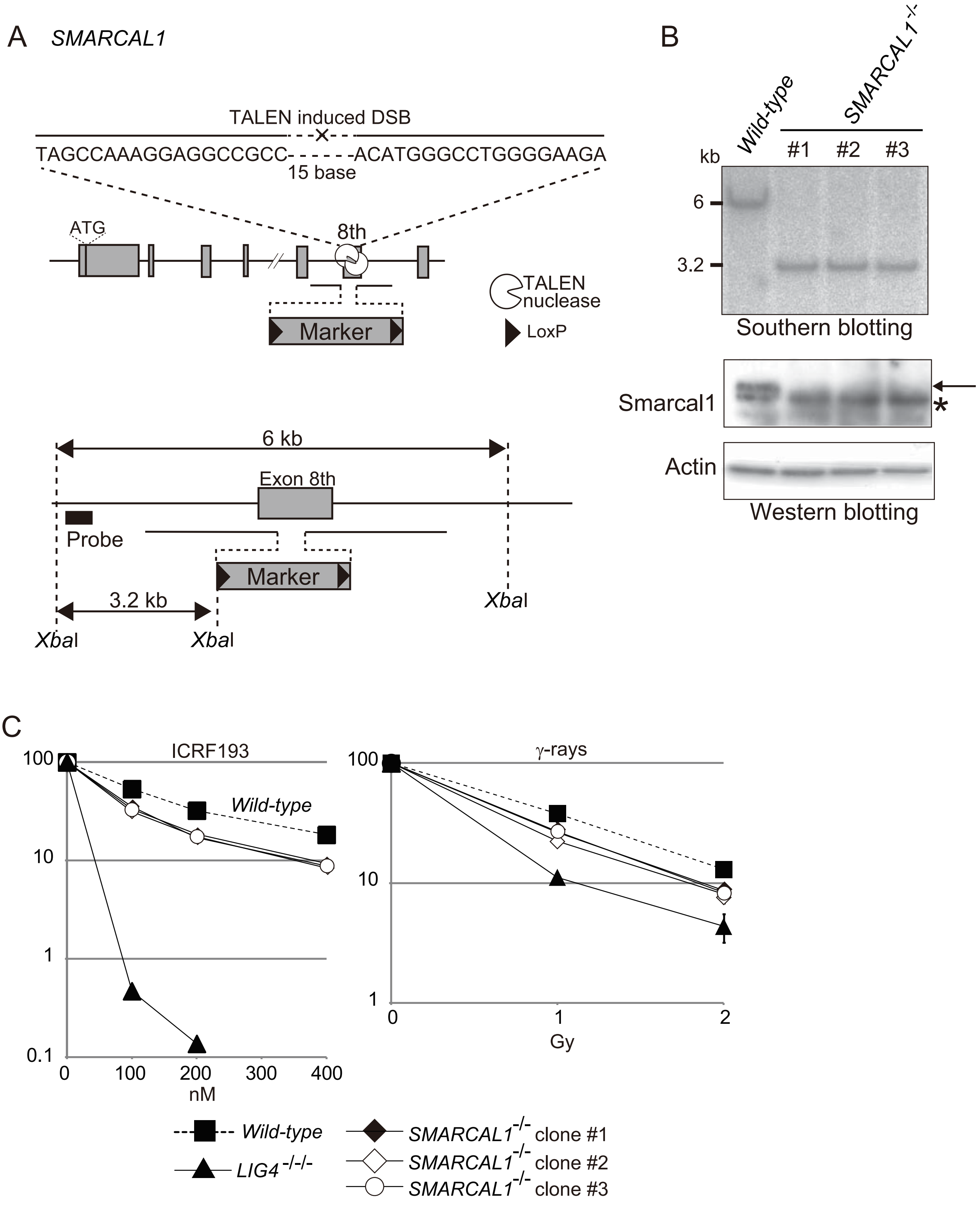

A
SMARCAL1
TALEN induced DSB
TAGCCAAAGGAGGCCGCC- - - - - -ACATGGGCCTGGGGAAGA
15 base
ATG
8th
TALEN nuclease
Marker
LoxP
6 kb
Exon 8th
Probe
Marker
3.2 kb
XbaI
XbaI
XbaI
B
Wild-type
SMARCAL1-/-
#1
#2
#3
kb
6
3.2
Southern blotting
Smarcal1
*
Actin
Western blotting
C
ICRF193
100
10
1
0.1
Wild-type
0
100
200
300
400
nM
γ-rays
100
10
1
0
1
2
Gy
Wild-type
LIG4-/-/-
SMARCAL1-/- clone #1
SMARCAL1-/- clone #2
SMARCAL1-/- clone #3

Keka, *et al*., Supplemental Figure 3

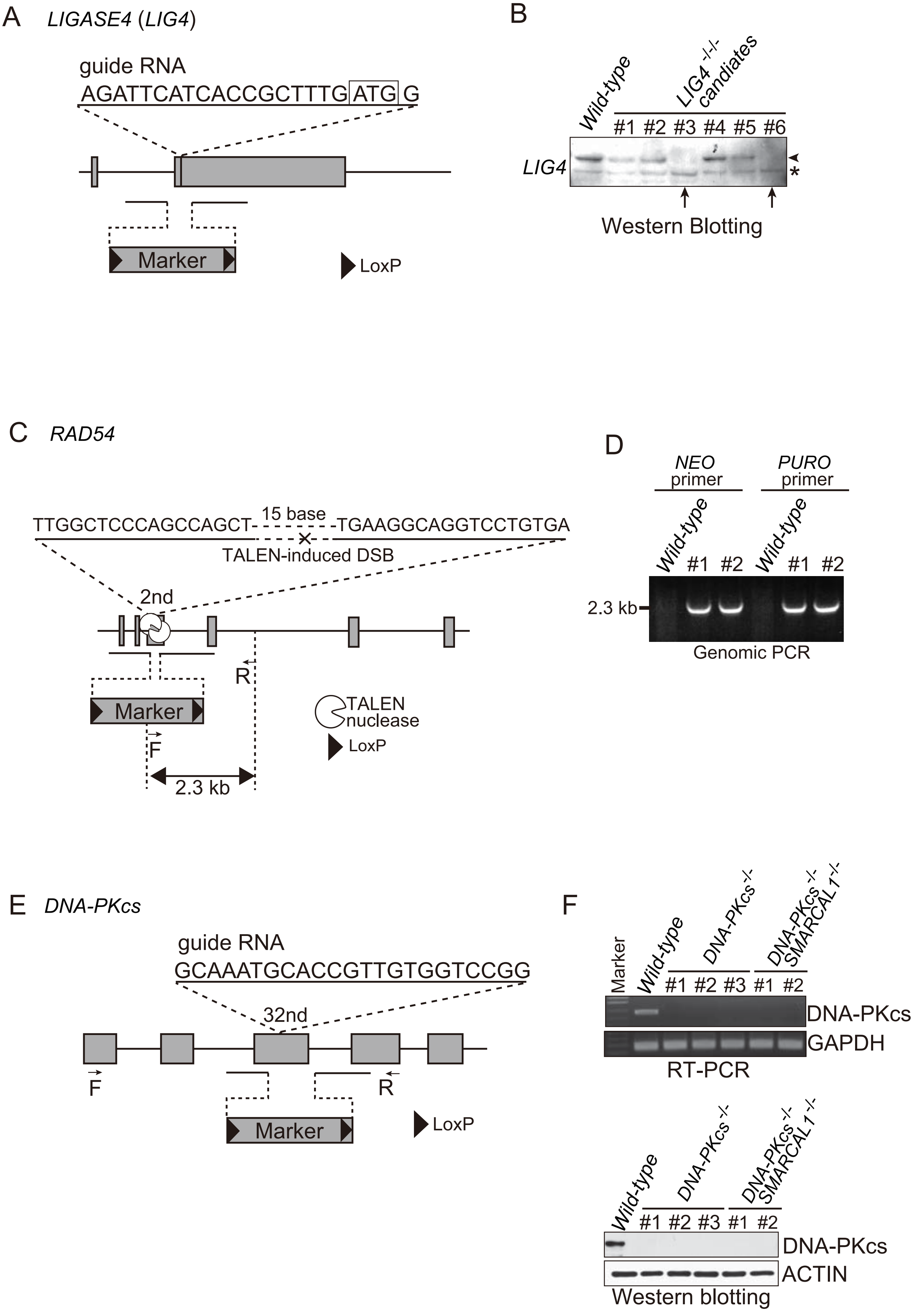

Keka, *et al.*, Supplemental Figure 4

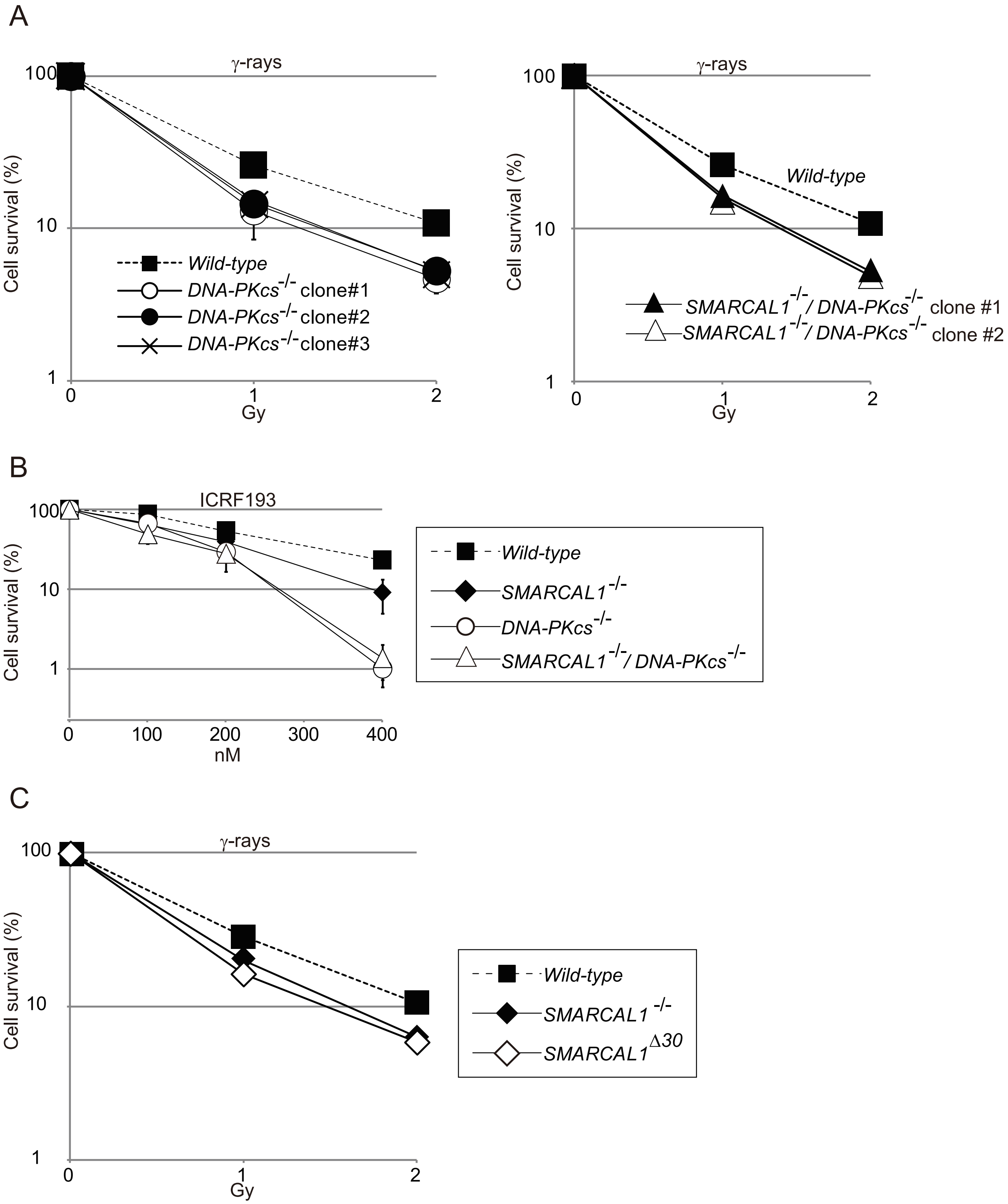

Keka, *et al*., Supplemental Figure 5

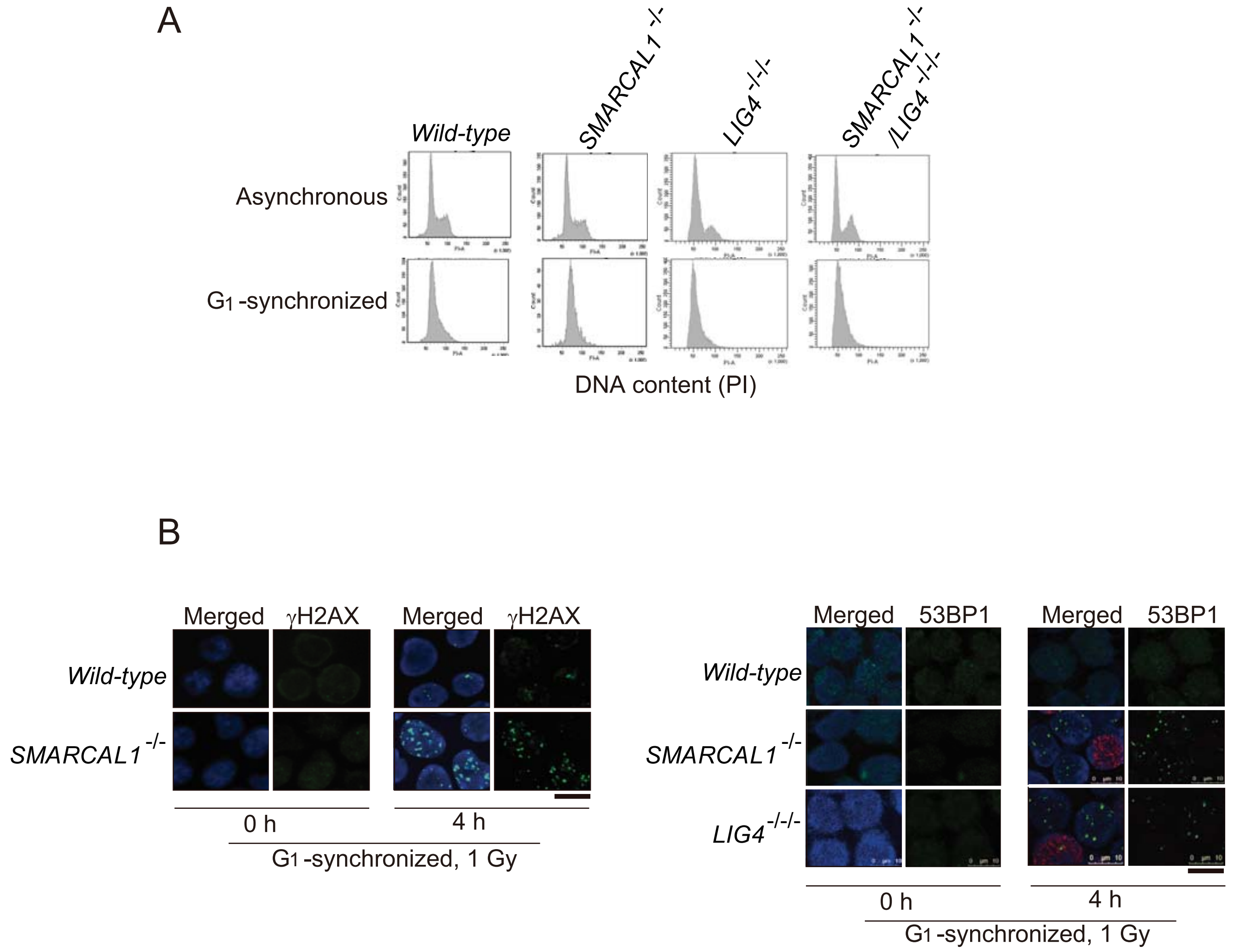

Keka., et al. Supplemental Figure 6

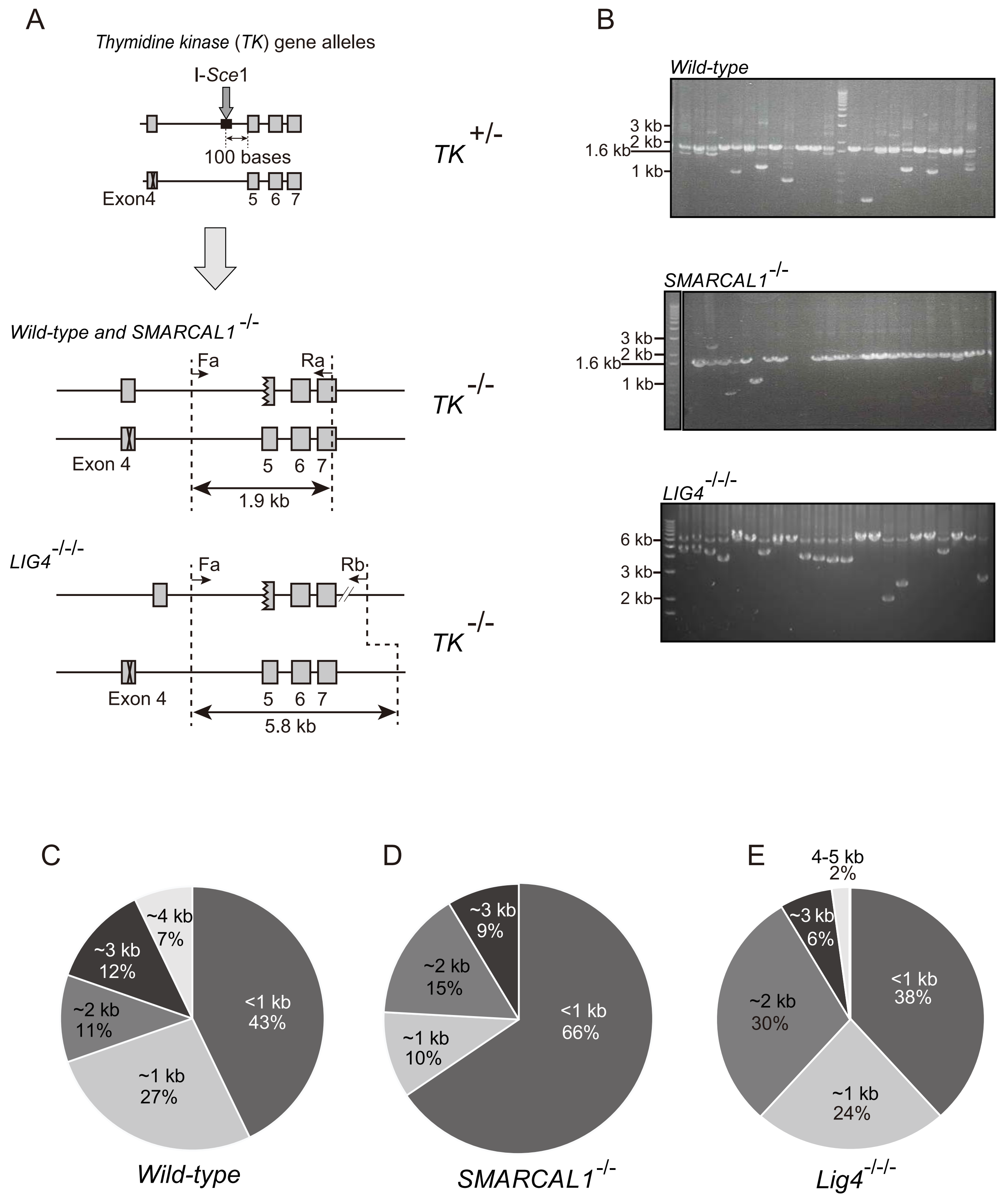

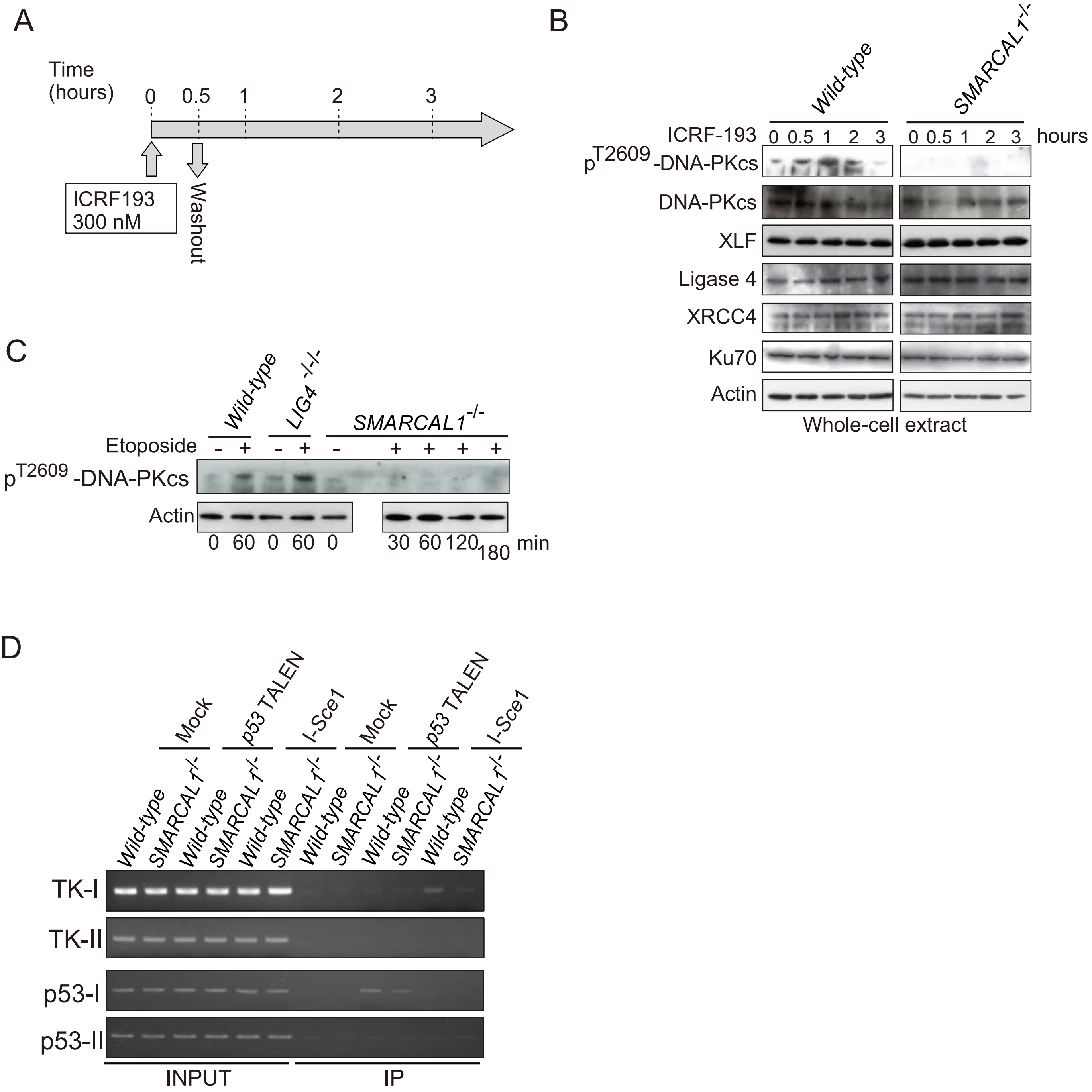
A
Time
(hours)
0
0.5
1
2
3
ICRF193
300 nM
Washout
B
Wild-type
SMARCAL1-/-
ICRF-193
0 0.5 1 2 3
0 0.5 1 2 3
hours
pT2609-DNA-PKcs
DNA-PKcs
XLF
Ligase 4
XRCC4
Ku70
Actin
Whole-cell extract
C
Wild-type
LIG4-/-/-
SMARCAL1-/-
Etoposide
- + - + - + + + +
pT2609-DNA-PKcs
Actin
0 60 0 60 0 30 60 120 180 min
D
Mock
p53 TALEN
I-Sce1
Mock
p53 TALEN
I-Sce1
Wild-type
SMARCAL1-/-
TK-I
TK-II
p53-I
p53-II
INPUT
IP

SUPPLEMENTAL MATERIALS AND METHODS

Generation of TALEN expression plasmids

TALEN-expression plasmids were constructed using the Platinum Gate TALEN Kit (Addgene) as previously described (doi: 10.1038/srep03379). A two-step Golden Gate reaction was performed to assemble DNA-binding repeats in the destination vector, ptCMV-153/47-VR, contained in the Platinum Gate TALEN Kit, except that the *p53* TALEN was assembled into a CAG promoter-driven vector.

Generation of human LIG4$^{-/-/-}$ TK6 B cells

The *LIG4* gene is located on chromosome 13, which is trisomic in TK6 cells (1). To disrupt the *LIG4* gene, we designed a guide RNA targeting the initiation codon using the Zhang CRISPR tool (2) and gene-targeting constructs. The CRISPR-target site is depicted in Figure S3A. The gene-targeting constructs were generated using SLiCE (Seamless Ligation Cloning Extract). The genomic DNA was amplified with primers F11 and R11 from the *LIG4*-gene locus and the PCR product was used as template DNA for amplifying the 5'- and 3'-arms. The 5'-arm was amplified using primers F12 and R12 and the 3'-arm was amplified using primers F13 and R13, where each primer shared 15-base pair-end homology with the insertion site of the vector. Both vectors, DT-ApA/neo and DT-ApA/puro, were linearized with *Afl*II and *Apa*I. All the fragments of the vectors and inserts were purified using a qiaquick gel extraction kit (QIAGEN, Netherlands). The gene-targeting constructs were generated in a single reaction mixture containing DT-ApA/neo or DT-ApA/puro vectors, 5'- and 3'-arms, and 2×SLiCE buffer (Invitrogen, US) and incubated for 30 min at room temperature. 6 μg of CRISPR and 2 μg of each gene-targeting vector were transfected into 4×10^6 TK6 cells using the Neon

Transfection System (Life Technologies, US). After electroporation, cells were released into 20 ml drug-free medium containing 10% horse serum. Forty-eight hours later, cells were seeded into 96-well plates for selection with both neomycin and puromycin antibiotics for two weeks. The *LIG4* gene-targeting event was confirmed by western blot analysis with anti-ligase4 antibody. The targeting efficiency of generating *LIG4*$^{-/-/-}$ clones from *LIG4*$^{+/+/+}$ cells was 10% (2/20).

Generation of human RAD54$^{-/-}$ *TK6 B cells*

To disrupt *RAD54*, TALEN-expression plasmids and gene-targeting constructs were generated. TALEN-target sites are depicted in Figure S3C. Gene-targeting constructs were generated from genomic DNA from TK6 cells by amplifying with primers *Xho*I-flanked F14 and *Nhe*I-flanked R14 for the 5'-arm and *Not*I-flanked F15 and *Sal*I-flanked R15 for the 3'-arm. The 5'-arm PCR product was cloned into the *Xho*I and *Nhe*I sites found upstream of *puro*^{R} and *neo*^{R} marker genes of the DT-ApA/puro and DT-ApA/neo vectors, respectively. The 3'-arm PCR product was cloned into the *Not*I and *Sal*I sites found downstream of the *puro*^{R} and *neo*^{R} marker genes of the DT-ApA/puro and DT-ApA/neo vectors, respectively. Six μg of TALEN expression plasmids and 2 μg of gene-targeting vector were transfected into 4×10^6 TK6 cells using Neon Transfection System (Life Technologies, US). After electroporation, cells were released into 20 ml drug-free medium containing 10% horse serum. Forty-eight hours later, cells were seeded into 96-well plates for selection with both neomycin and puromycin antibiotics for two weeks. The genomic DNA of clones resistant to both neomycin and puromycin was amplified using primers F16 (designed inside *neo*^{R} genes), F17 (designed inside *puro*^{R} genes), and R16 (designed outside of the 3'-arm) to confirm the disruption of *RAD54* gene. The targeting efficiency of generating *RAD54*$^{-/-}$ clones from *RAD54*$^{+/+}$ cells was

20% (2/10).

Phosphorylation analysis of DNA-PKcs at Thr2609

TK6 cells were treated with 300 nM of ICRF193 or 30 nM of etoposide and released 30 min later into drug-free media. Cells were harvested at time-0 min (without drug treatment), 30 min, 1 h, 2 h, and 3 h. Cells were lysed using 2× sample buffer and whole-cell extracts (WCE) were used for western blot analysis.

Antibodies

Anti-DNA-PKcs phosphorylated (Thr2609) mouse monoclonal (#612901, BioLegend, US); anti-DNA-PKcs (#612701, BioLegend, US) mouse monoclonal; anti-Smarcal1 rabbit polyclonal (ab154226, abcam, UK), the recombinant fragment corresponding to a region within amino acids 120 and 457 of Human Smarcal1; anti-XRCC4 goat polyclonal (C-20, Santa Cruz, US); anti-XLF rabbit polyclonal (#2854, Cell Signaling, US).

Supplemental Figure legends

Figure S1. Generation of *SMARCAL1* mutants from chicken DT40 cells. (A) Schematic representation of the *SMARCAL1* locus (upper) and configuration of the targeting construct carrying a marker gene (lower) flanked by ~1 kb genomic sequences on both sides. Open boxes represent exon sequences. Note that the size of the schematic representation does not reflect the actual size of the DNA. (B) Southern blot analysis showing the deletion of both allelic *SMARCAL1* genes. Genomic DNA was digested with both *Sma*I and *Xho*I and hybridized with the probe indicated in (A). (C) RT-PCR with the primers indicated in (D) confirmed the inactivation of the *SMARCAL1* gene in *SMARCAL1*$^{-/-}$ cells. (D) Structure of the intact *SMARCAL1* allele in *SMARCAL1*$^{+/-}$ cells and the Δ30 deletion construct (top), including genomic fragments and a selection marker gene with *loxP* signals (closed triangles). RT-PCR with the indicated primer set yielded a 400 bp band. Targeting of the Δ30 deletion construct into the intact allele changed its structure to the one indicated in the *SMARCAL1*$^{-/-}$ cells. Transient expression of cre-recombinase in *SMARCAL1*$^{-/-}$ cells popped out the marker gene, leading to the generation of *SMARCAL1*$^{\Delta 30/-}$ cells. The pair of opposing arrows indicates the primers for RT-PCR. RT-PCR of transcripts from the intact *SMARCAL1* and *SMARCAL1*$^{\Delta 30}$ alleles generated 400 bp and 310 bp products, respectively. (E) RT-PCR data with the primers shown in (D) indicated the expression of Smarcal1 lacking 30 amino acids at the N-terminus.

Figure S2. Generation of *SMARCAL1*$^{-/-}$ human TK6 cells. (A) Schematic

representation of the human *SMARCAL1* locus, base sequences of TALEN-recognition sites, and structure of the targeting constructs containing genomic fragments and a selection-marker gene (*hygro*^{R} or *puro*^{R}) with *loxP* signals on both sides. The cross indicates the TALEN-induced DSB site. A pair of TALEN was designed to target exon 8 (corresponding to 446-495 amino acids of Smarcal1), which contains the DEXDc domain. The lower panel is a magnification of the upper panel. (B) Southern blot analysis of three *SMARCAL1*$^{-/-}$ clones (upper panel) shows deletion of *SMARCAL1* genes. Genomic DNA was digested with *Xba*I and hybridized with the probe indicated in (A). Western blot analysis for Smarcal1 protein (lower panel). The horizontal arrow indicates Smarcal1-protein expression and the asterisk indicates a non-specific band. β-actin is used as a loading control. (C) Clonogenic-cell-survival assay following exposure of the indicated genotypes to DNA-damaging agents. The x-axis represents the dose of the indicated DNA-damaging agent on a linear scale; the y-axis represents the survival fraction on a logarithmic scale. Error bars show the SD of the mean for three independent assays.

Figure S3. Generation of *LIG4*$^{-/-/-}$, *RAD54*$^{-/-}$ and *DNA-PKcs*$^{-/-}$ human TK6 cells. (A) Schematic representation of the human *LIG4* locus, target sequences by CRISPR/Cas9, and the gene-targeting construct with a selection marker. The box around ATG indicates the initiation codon. (B) Western blot analysis showing Ligase4 protein expression in *wild-type* cells and six clones transfected with the gene-targeting construct and CRISPR/Cas9. Clones #3 and #6, indicated by arrows, were identified as *LIG4*$^{-/-/-}$ cells. The arrowhead indicates Ligase4 protein and the asterisk indicates a non-specific band. (C) Schematic representation of the human *RAD54* locus and configuration of the

targeted allele. The *RAD54*-disrupting constructs contain the *neo*^{R} or *puro*^{R} marker gene. A pair of TALEN was designed to target exon2. The TALENs together with the two gene-targeting constructs were transfected, and TK6 clones resistant to both neomycin and puromycin were analyzed by genomic PCR. (D) Genomic PCR analysis of *wild-type* and two *RAD54*$^{-/-}$ clones using the indicated forward (F) and reverse (R) primers. The three lanes on the left and three lanes on the right include PCR products using neomycin- and puromycin-resistance-gene-specific forward primers and a reverse primer designed outside of the 3'-arm, respectively. (E) Schematic representation of the human *DNA-PKcs* locus, target sequences by CRISPR/Cas9, and the gene-targeting construct with a selection marker. (F) RT-PCR analysis (upper panel) using the forward (F) and reverse (R) primers indicated in (E), confirmed the inactivation of the *DNA-PKcs* gene in *DNA-PKcs*$^{-/-}$ cells and *DNA-PKcs*$^{-/-}$ *SMARCAL1*$^{-/-}$ cells. GAPDH is used as a loading control. Western blot analysis (lower panel) showing DNA-PKcs protein expression only in the *wild-type* cells, but not in the five clones transfected with the gene-targeting construct and CRISPR/Cas9. All the clones were identified as *DNA-PKcs*$^{-/-}$ cells. β -actin is used as a loading control.

Figure S4. *DNA-PKcs*$^{-/-}$, *SMARCAL1*$^{-/-}$ and *SMARCAL1*$^{-/-}$/*DNA-PKcs*$^{-/-}$ TK6 B cells are sensitive to ICRF193 and γ-rays. Clonogenic-cell-survival assay following exposure of the indicated genotypes to DNA-damaging agents (A–C). The x-axis represents the dose of the indicated DNA-damaging agent on a linear scale; the y-axis represents the survival fraction on a logarithmic scale. Error bars show the SD of the mean for three independent assays.

Figure S5. γ-ray-induced DNA-damage response in TK6 cells at the G_1 phase. (A) DNA content of G_1-synchronized TK6 cells of the indicated genotypes. (B) Representative images show γH2AX foci (green) and 53BP1 foci (green) in the indicated TK6 genotypes synchronized at the G_1 phase. Merged images show DAPI (blue) and EdU (red) staining. The horizontal bar in the bottom-right corner is a 10 μm scale bar.

Figure S6. Analysis of deletion range at the I-*Sce*1 induced DSB sites. (A) Schematic diagram showing nucleotide-sequence loss from the I-*Sce*1-induced DSB site in the *wild-type* allele of cells heterozygous for the thymidine kinase (*TK*) gene. Deletion of more than 100 nucleotides during DSB repair inactivates exon5 of the *wild-type TK* allelic gene, leading to the formation of $TK^{-/-}$ clones from $TK^{+/-}$ cells. The pair of opposing horizontal arrows indicates the primers for measuring the length of deletion in $TK^{-/-}$ clones. We used the Rb primer for the analysis of $LIG4^{-/-/-}$cells, since some clones had deletion beyond the Ra primer. (B) Gel images show the 1.6 kb band (*wild-type* and $SMARCAL1^{-/-}$ cells) and 5.8 kb band ($LIG4^{-/-/-}$cells) derived from the intact *TK* allele along with different sized bands representing deletion events. (C-E) Pie charts depict the percentage of different length deletions from the I-*Sce*1 site. The detail of this experiment has been described previously (3).

Figure S7. Smarcal1 is required for the phosphorylation of DNA-PKcs at threonine 2609. (A) Experimental design. TK6 cells were treated with 300 nM ICRF193 for 30 minutes and harvested for western blot analysis at the indicated time points. (B) Western blot analysis to detect phosphorylation at threonine 2609 of DNA-PKcs, and for

DNA-PKcs, Ligase4, XLF, XRCC4 and Ku70 proteins at the indicated time as shown in (A). β-actin is used as a loading control. (C) Western blot analysis to measure DNA-PKcs phosphorylation at threonine 2609. The experiment was done as shown in (A), except that cells were treated with 30 nM etoposide instead of ICRF193. (D) Gel image for PCR analysis of the input and immunoprecipitated DNA fragments from the Figure 6 D-G.

Supplemental Table 1. Primer list.

F1	5’-GCATTCACTTTGATATCCTTTTGGATCAAA-3’
R1	5’-GAAATATCACTTACCTCATCAGCAATCATA-3’
F2	5’-TTCTGTGAATATTCACTTCCTTATAGCGAA-3’
R2	5’-TGCTTATTTCAGCTTTACCTTTGTATAGAG-3’
F3	5’-TTCTtTGAGTTATTCCCATCAACATTACTC-3’
R3	5’-GGTTGGTATCTTCTTAAACAAAGCAATAAG-3’
F4	5’-TAAAAAATGGACGTTTACTTCTTGCGGATG-3’
R4	5’-AATAACTACTTGGAAAGTGCTCTTGAGTTG-3’
F5	5’-GGCGGCCGCTACAAGTTCTCTCCATATGGTTGAAGACTA-3’
R5	5’-GGGATCCCAATTCAAACAAACCTGTGTCAGTAC-3’
F6	5’-GGGATCCATGAGCAAGGTGGGCAGCTGGGGATCACTGGGCC-3’
R6	5’-GGTCGACAGAAAATGCAGTTGACTTGTCATCAGGCTC-3’
F7	5’-AGGTTAAATCATGT-3’
R7	5’-CAAGGTGAGGATGG-3’
F8	5’-CCATCCAAGCCATCTGC-3’
R8	5’-CTAGAAAAAGCTGTGGA-3’
F9	5’-TACATAGCACACTCTGCAAAACCTCCTCTG-3’
R9	5’-CTTCTGACTGCTGAATAACTTTAGGCAACT-3’
F10	5’-GGATCCATGCATCAGAGGACTAGCTCGGGCAC-3’
R10	5’-AAGCTTTTACAG GGGAGACGTAAAGCTGTCCCA-3’
F11	5’- ATAGTAAATGTAGTATTGTAATTAGCCATTT-3
R11	5’- GTCCAATTCATCCATTAGTCCACTGACATA-3’
F12	5’- GCGAATTGGGTACCGGGCCATAGTAAATGTAGTA-3’

R12	5’- CTGGGTCGAGGGGGGGCCATGCTCTAAATGATG-3’
F13	5’- TGGGAAGCTTGTCGACTTAATTGGAGAAAATTTCA-3’
R13	5’- CACTAGTAGGCGCGCCTTAAGTCCAATTCATCCAT-3’
F14	5’-CCTCGAGCCAGCTGTCCACTACAGGTGGTAACTC-3’
R14	5’-GGCTAGCAGCTCCTCCTCTGTAAAGGGAGAACAG-3’
F15	5’-GCGGCCGCAACCTGGCCTAGTGGTGAGCACTCAAG-3’
R15	5’-GGTCGACCAGTTCTGGCTTACATGCTGACTGCTG-3’
F16	5’-AACCTGCGTGCAATCCATCTTGTTCAATGG-3’
R16	5’-ATGTGTGTCTTCTGGCCACTCTAGAAAGCA-3’
F17	5’-GTGAGGAAGAGTTCTTGCAGCTCGGTGA-3’
R17	5’-CTTTCATTGCCATACG-3’
F18	5’-ACCTCATCAGCTGAGCAGGAGGACCTGTGCCAGCA-3’
R18	5’-TGCTGGCACAGGTCCTCCtGCTCAGCTGATGAGGT-3’
F19	5’- ATAAGTTATCTCAGTTCTTGGAATACATCA-3’
R19	5’- TCACACAAACATCAGGAAGATGAGCCATAA-3’
F20	5’- GCGAATTGGGTACCGGGCCATAAGTTATCTCAGTTCTT-3’
R20	5’- CTGGGCTCGAGGGGGGGCCTTGTGGGCTTGTTCTGTTACC-3’
F21	5’- TGGGAAGCTTGTCGACTTAAGCTGCTCTGTTAATTTGAGG-3’
R21	5’- CACTAGTAGGCGCGCCTTAATCACACAAACATCAGGAAG-3’
F22	5’- AAGGTGTCTCTTTTCTCATCAACACCTTTG-3’
R22	5’- GGATATCTTTGTATGGGGACATCTTTAGAGC-3’
Fa	5’-CTGGGAGCTGCATTTTCGTGACATGTCATG-3’
Ra	5’-TGCATTGCAGAATCTGCTGTGGGGCAAAGA-3’
Rb	5’-CGTGTTCTCCTGCATCTATGGTGAGGGCTA-3’
TK-IF	5’-TTTCCAGTTCCCTGACATCGTGGAG-3’
TK-IR	5’-ATCCAGAAGCCTCTTGCCCTCCTCAAT-3’
TK-IIF	5’-GCTTTCACTGCTGAGTTTCTGTTCTCCCTG-3’
TK-IIR	5’-GTAGGAAGGGCTTTGAGCCCAAAAGGAAAC-3’
p53-IF	5’-AAGTAAATGGGTTTAACTATTGCACAGTTG-3’
p53-IR	5’-TTTTGTATTTTTCAGTAGAGACGGGGTTTC-3’
p53-IIF	5’-TTTTAGTAGAGATGAGGTTTCACCATGTTG-3’
p53-IIR	5’-GAGGGTATAATGAGCTATGATCACATCACT-3’

Supplemental Table 2. Sequence analysis of pJH290 recombinant plasmids.

**Correct joint**	**CCAAGCTTGGGCTGCAGGTCGAC**	**GGATCCCCGGGGATCAGCTT**	**No.**
WT	CCAAGCTTGGGCTGCAGGTCG	ATCCCCGGGGATCAGCTT	4
	CCAAGCTTGGGCTGCAGG	ATCCCCGGGGATCAGCTT	7
	CCAAGCTTGGGCTGCAGG	ATCCCCGGGGATCAGCTT	7
	CCAAGCTTGGGCTGCAGG	ATCCCCGGGGATCAGCTT	7
	CCAAGCTTGGGCTGCAGGTCGA	CCCCGGGGATCAGCTT	5
	CCAAGCTTGGGCTG	CCCGGGGATCAGCTT	14
	CCAAGCTTGGGCTGCAGGTC	CCCGGGGATCAGCTT	8
	CCAAGCTTGGGCTGCAGGTCGAC	GGATCCCCGGGGATCAGCTT	0
	CCAAGCTTGGGCTGCA	GGATCCCCGGGGATCAGCTT	7
	CCAAGCTTGGGCTGCAGGTC	CCCGGGGATCAGCTT	8
	CCAAGCTTGGGCTGCA	GGATCCCCGGGGATCAGCTT	7
	CCAAGCTTGGGCTGCAGGTCGAC	CCGGGGATCAGCTT	6
	CCAAGCTTGGGCTGCAG	GATCCCCGGGGATCAGCTT	7
	CCAAGCTTGGGCTGCAG	GATCCCCGGGGATCAGCTT	7
	CCAAGCTTGGGCTGCA	GATCCCCGGGGATCAGCTT	6
	CCAAGCTTGGGCTGCAG	ATCCCCGGGGATCAGCTT	6
SMARCAL1$^{-/-}$	CCAAGCTTGGGCTGCAG	ATCCCCGGGGATCAGCTT	8
	CCAAGCTTGGGCTGCAGGTCGA	CCCGGGGATCAGCTT	6
	CCAAGCTTGGGCTGCAGG	ATCCCCGGGGATCAGCTT	7
	CCAAGCTTGGGCTGCAGG	ATCCCCGGGGATCAGCTT	7
	CCAAGCTTGGGCTGCAGG	ATCCCCGGGGATCAGCTT	7
	CCAAGCTTGGGCTGCAGGTCG	CCCCGGGGATCAGCTT	6
	CCAAGCTTGGGCTGCAGG	CCCGGGGATCAGCTT	10
	CCAAGCTTGGGCTGCAGG	ATCCCCGGGGATCAGCTT	7
	CCAAGCTTGGGCTGCAGGT	ATCCCCGGGGATCAGCTT	6
	CCAAGCTTGGGCTGCAGGT	CCCCGGGGATCAGCTT	8
	CCAAGCTTGGGCTGCAGGT	CCCCGGGGATCAGCTT	8

	CCAAGCTTGGGCTGCAGG	CCCGGGGATCAGCTT	10
	CCAAGCTTGGGCTGCAGGT	CCCCGGGGATCAGCTT	8
	CCAAGCTTGGGCTGCAGGT	CCCCGGGGATCAGCTT	8
	CCAAGCTTGGGCTGCAGGTCG	ATCCCCGGGGATCAGCTT	4
	CCAAGCTTGGGCTGCAGGTCGAC	ATCCCCGGGGATCAGCTT	2
	CCAAGCTTGGGCTGCAG	GATCCCCGGGGATCAGCTT	7
	CCAAGCTTGGGCTGCAGGTCG	CCCCGGGGATCAGCTT	6
	CCAAGCTTGGGCTGCAGGTCG	ATCCCCGGGGATCAGCTT	4
	CCAAGCTTGGGCTGCAG	GGATCCCCGGGGATCAGCTT	6
	CCAAGCTTGGGCTGCAGG	TCCCCGGGGATCAGCTT	8
LIG4$^{-/-/-}$	CCAAGCTTGGGCTGCAGGT	GATCCCCGGGGATCAGCTT	5
	CCAAGCTTGGGCTGCAGGTCG	ATCCCCGGGGATCAGCTT	4
	CCAAGCTTGGGCTGCAGGT	GGATCCCCGGGGATCAGCTT	4
	CCAAGCTTGGGCTGCAG	GATCCCCGGGGATCAGCTT	7
	CCAAGCTTGGGCT	ATCCCCGGGGATCAGCTT	12
	CCAAGCTTGGGCTGCAGGTC	GGATCCCCGGGGATCAGCTT	3
	CCAAGCTTGGGCTGCAGGTCG	CCCCGGGGATCAGCTT	6
	CCAAGCTTGGGCTGCAGG	TCCCCGGGGATCAGCTT	8
	CCAAGCTTGGGCTGCAGGT	ATCCCCGGGGATCAGCTT	6
	CCAAGCTTGGGCTGCAGGTCGAC	GGATCCCCGGGGATCAGCTT	+1
	CCAAGCTTGGGCTGCA	GGATCCCCGGGGATCAGCTT	7

Coding-end sequences as they occur in the pJH290 substrate are shown in sequences of the recombinant junctions. The number of bases deleted in each sequence is shown in the right-hand column. All duplicate sequences were derived from separate transfections.

REFERENCES

1. Grosovsky, A.J., Parks, K.K., Giver, C.R. and Nelson, S.L. (1996) Clonal analysis of delayed karyotypic abnormalities and gene mutations in radiation-induced genetic instability. *Molecular and cellular biology*, **16**, 6252-6262.
2. Ran, F.A., Hsu, P.D., Lin, C.Y., Gootenberg, J.S., Konermann, S., Trevino, A.E., Scott, D.A., Inoue, A., Matoba, S., Zhang, Y. *et al.* (2013) Double nicking by RNA-guided CRISPR cas9 for enhanced genome editing specificity. *Cell*, **154**,

1380-1389.
3. Honma, M., Izumi, M., Sakuraba, M., Tadokoro, S., Sakamoto, H., Wang, W., Yatagai, F. and Hayashi, M. (2003) Deletion, Rearrangement, and Gene Conversion; Genetic Consequences of Chromosomal Double-Strand Breaks in Human Cells. *Environmental and molecular mutagenesis*, **42**, 288-298.
